# Analysis of global Aeromonas caviae genomes revealed that strains carrying T6SS are more common in human gastroenteritis than in environmental sources and are often phylogenetically related

**DOI:** 10.1099/mgen.0.001258

**Published:** 2024-05-30

**Authors:** Sarah K. T. Chong, Fang Liu, Christopher Yuwono, Alfred Chin Yen Tay, Michael C. Wehrhahn, Stephen M. Riordan, Lu Liu, Li Zhang

**Affiliations:** 1 School of Biotechnology and Biomolecular Sciences, University of New South Wales, Sydney, Australia; 2 Helicobacter Research Laboratory, School of Pathology and Laboratory Medicine, Marshall Centre for Infectious Diseases Research and Training, University of Western Australia, Perth, Australia; 3 Douglass Hanly Moir Pathology, Macquarie Park, New South Wales, Australia; 4 Gastrointestinal and Liver Unit, Prince of Wales Hospital, University of New South Wales, Sydney, Australia; 5 School of Biomedical Sciences, University of New South Wales, Sydney, Australia

**Keywords:** *Aeromonas*, *Aeromonas caviae*, gastroenteritis, genome, secretion system, toxins

## Abstract

*Aeromonas caviae* is an emerging human enteric pathogen. However, the genomic features and virulence genes of *A. caviae* strains from human gastroenteritis and other sources have not been fully elucidated. Here, we conducted a genomic analysis of 565 global *A. caviae* strains isolated from different sources, including 261 strains isolated from faecal samples of gastroenteritis patients, of which 18 genomes were sequenced in this study. The presence of bacterial virulence genes and secretion systems in *A. caviae* strains from different sources was compared, and the phylogenetic relationship of *A. caviae* strains was assessed based on the core genome. The complete genome of *A. caviae* strain A20-9 isolated from a gastroenteritis patient was obtained in this study, from which 300 putative virulence factors and a T4SS-encoding plasmid, pAC, were identified. Genes encoding T4SS were also identified in a novel genomic island, ACI-1, from other T4SS-positive strains. The prevalence of T4SS was significantly lower in *A. caviae* strains from gastroenteritis patients than in environmental strains (3 %, *P*<0.0001 vs 14 %, *P*<0.01). Conversely, the prevalence of T6SS was significantly higher in *A. caviae* strains isolated from gastroenteritis patients than in environmental strains (25 %, *P*<0.05 vs 13  %, *P*<0.01). Four phylogenetic clusters were formed based on the core genome of 565 *A*. *caviae* strains, and strains carrying T6SS often showed close phylogenetic relationships. T3SS, aerolysin and thermostable cytotonic enterotoxin were absent in all 565 *A*. *caviae* strains. Our findings provide novel information on the genomic features of *A. caviae* and suggest that T6SS may play a role in *A. caviae*-induced human gastroenteritis.

**Impact Statement**

*Aeromonas caviae* has been gaining attention as an emerging human pathogen for causing several diseases in infected individuals, with gastroenteritis being one of the most common clinical manifestations. Diseases caused by *Aeromonas* infections are a global concern, with literature from different countries reporting their incidence. Despite its clinical significance, there is limited information on how the bacterial genomic features of *A. caviae* strains isolated from human gastroenteritis compare to strains from other sources. Here, we conducted a genomic analysis of 565 global *A. caviae* strains isolated from different sources and examined the presence of virulence factors. We identified a novel *A. caviae* plasmid, a T4SS genomic island, four phylogenetic clusters of *A. caviae* strains based on the core genome, and a positive association between T6SS and *A. caviae* strains isolated from gastroenteritis patients. Our findings provide novel information on the genomic features of *A. caviae* and suggest the role of T6SS-carrying *A. caviae* strains in causing human gastroenteritis.

## Data Availability

The genome assemblies of the 18 *A. caviae* genomes sequenced in this study have been deposited in the NCBI bacterial genome database and the raw reads have been deposited to the SRA database under the BioProject PRJNA1050808.

## Introduction

*Aeromonas* species are gram-negative facultative anaerobic bacteria that are ubiquitous in aquatic environments [1]. In addition to their role as fish pathogens, several *Aeromonas* spp. have emerged as human enteric pathogens, often causing gastroenteritis in infected individuals [2]. While most patients may recover without medical treatment, patients with more severe infections require antibiotic therapy or hospital admission [3]. Other human diseases that may arise from infections with *Aeromonas* spp. include bacteraemia, wound infection, skin and soft tissue infection, pneumonia, and biliary tract infection [3,5].

A recent Australian study revealed that *Aeromonas* spp. exhibited a distinct infection pattern in human gastroenteritis compared to other enteric bacterial pathogens [6]. Across all age groups, three infection peaks occurred in young children under four, young adults aged 20–29, and adults over 50. *Aeromonas* spp. were most commonly identified in children under 18 months, with infants between 6–12 months old exhibiting the highest infection rate at 607 per 10 000 samples. In contrast, infection rates of *Campylobacter* spp., *Salmonella* spp., *Shigella* spp./enteroinvasive *Escherichia coli* and *Yersinia* spp. in the same age group were 320, 294, 19 and 42 per 10 000 samples, respectively [6]. Additionally, patients with inflammatory bowel disease (IBD) are more likely to be infected with *Aeromonas* spp. than patients without IBD [7].

Several *Aeromonas* spp. are associated with human gastroenteritis, with *A. caviae* being frequently reported along with *Aeromonas veronii* and *Aeromonas hydrophila* [2]. In Pakistan and Bangladesh, *A. caviae* is the most commonly isolated *Aeromonas* spp. in children under 5 years of age [8]. Despite its significance in causing human gastrointestinal infections, the genomic features and virulence genes of *A. caviae* strains isolated from gastroenteritis patients in comparison to strains isolated from other sources have not been fully elucidated.

To address these knowledge gaps, we conducted a genomic analysis of 565 global *A. caviae* strains isolated from different sources, including 261 strains isolated from faecal samples of gastroenteritis patients, 58 strains from patients with diseases other than gastroenteritis and 246 strains from non-human sources. Our study provides novel findings on the genomic features of *A. caviae* and the association of type 6 secretion system (T6SS)-positive *A. caviae* strains with human gastroenteritis.

## Methods

### Draft genome sequencing of 18 *A. caviae* strains and complete genome sequencing of *A. caviae* strain A20-9

The sequencing and assembly of draft genomes of 18 *A*. *caviae* strains were conducted using Illumina MiSeq as described in our previous study [9]. These strains were isolated from faecal samples of gastroenteritis patients during routine diagnostic procedures at the Douglass Hanly Moir Pathology Laboratory in Sydney, Australia. The DNA libraries were sequenced via 150 bp or 250 bp paired-end sequencing chemistry on the MiSeq Personal Sequencer [10]. Reads were assembled using Shovill (v 1.0.5) (github.com/tseemann/shovill) and genome coverage was calculated using qualimap (v 2.1.11) [11,12]. Draft genome sequencing was performed by the Marshall Centre for Infectious Diseases Research at the University of Western Australia.

The genome of *A. caviae* A20-9 was randomly selected for further sequencing by the Ramaciotti Centre for Genomics at the University of New South Wales using the Oxford Nanopore sequencing technique. Bacterial DNA was first extracted with phenol-chloroform [13]. DNA libraries were then prepared using the Native Barcoding Expansion Kit (EXP-NBD112, Nanopore) and Ligation Sequencing Kit (SQK-NBD112-24, Nanopore). The libraries were loaded onto an R10.4 flow cell (FLO-MIN112) and sequenced on the GridION sequencing device (Nanopore). Basecalling was performed using Guppy (v 6.1.2) (github.com/nanoporetech/rerio). Nanopore reads were filtered using Filtlong (github.com/rrwick/Filtlong), removing reads less than 1 000 bp and retaining 90 % of the highest-quality reads. Low-quality reads were removed until 500 Mbp remained. Filtered Nanopore reads were assembled using Flye (v 2.9.0) [14]. To obtain the complete genome of *A. caviae* A20-9, assemblies of Nanopore and Illumina MiSeq reads were combined for hybrid assembly using Unicycler (v 0.4.7) [15]. Statistics of the reads were generated using NanoStat (v 1.5.0), and genome coverage was estimated using Minimap2 (v 2.17) and qualimap (v 2.1.11) [11,16, 17]. A circular representation of the A20-9 plasmid was generated using GenoVi [18]. Plasmid similarity was searched against the NCBI database using blastn. Plasmids with at least 90 % query coverage from completely sequenced genomes were determined as similar.

### Total *A. caviae* genomes analysed in this study

A total of 565 *A*. *caviae* genomes were analysed in this study, including 18 genomes sequenced in this study and 548 from public databases. Currently, there are 247 genomes available in the NCBI genome database and 618 strains with Illumina paired-end raw reads in the Sequence Read Archive (SRA) database. Illumina raw reads were obtained from the SRA database from NCBI and were assembled using Shovill (v 1.1.0) (github.com/tseemann/shovill) [19]. Information on these strains was screened to exclude strains from unspecified sources, human health conditions, or poor genome quality. The quality of the genome assemblies was assessed by gene completeness ≥95 % and contamination ≤5 % using CheckM (v 1.2.2), and a criterion which requires a total assembly length between 4 and 5.8 Mb, N50 of ≥20 kb, ≤600 contigs and ≥70 % sequence reads assigned to the correct species. Additionally, the ANI values were compared among all strains, only selecting strains with ≥96 % similarity [20,23].

The genomes of the 565 *A*. *caviae* strains examined in this study were annotated with Rapid Annotation using Subsystem Technology (rast) and prokka (v 1.14.5) [24,25]. Of the 565 *A*. *caviae* strains, 261 were isolated from faecal samples of gastroenteritis patients. Information on the genomic data and isolation sources of all 565 *A*. *caviae* strains is summarized in Table S1, available in the online version of this article.

### Identification of virulence factors and secretion systems in *A. caviae* strains

Putative virulence factors in the complete genome of *A. caviae* A20-9 were identified using the Virulence Factors Database (VFDB) [26].

The presence of toxins in all 565 *A*. *caviae* strains examined in this study was determined using blastp, and conserved protein motifs were confirmed using InterPro [27,28]. These toxins have previously been reported in different *Aeromonas* spp. [29]. The protein sequences of aerolysin, haemolysin family protein, haemolysin III, thermostable haemolysin, microbial collagenase, and zonula occludens toxin (Zot) used as queries for blastp were obtained from *A. veronii*, thermostable cytotonic enterotoxin (Ast) and lipase (Alt) from *A. hydrophila*, and haemolysin D from *A. caviae*. The protein sequence of haemolysin D was retrieved from NCBI, whereas the protein sequences of the other toxins were obtained from a previously published paper [30].

The prevalence of secretion systems of 565 *A*. *caviae* strains was examined. prokka-annotated protein files were submitted to MacSyFinder (v 2.1.2) to identify all possible secretion system proteins using default search settings [31]. The visualization of type 4 secretion system (T4SS) was performed using EasyFig [32]. The nucleotide sequences of *A. caviae* T4SS were searched against the genomes of all the bacterial strains in the NCBI nonredundant nucleotide database using blastn [27]. The isolation sources of all 565 strains were categorized as human gastroenteritis samples, other human samples, animal samples and environmental samples. The group ‘Animals’ consisted of strains isolated from guinea pigs, a zebu, a dog, zooplankton, freshwater fish, and mussels. The group ‘Environment’ consisted of strains isolated from the natural environment, hospital environment, farm environment, wastewater, brackish water, freshwater, and seawater.

### Phylogenetic analysis of *A. caviae* genomes

Core-genome alignments were generated with Roary (v 3.12.0) for all 565 *A*. *caviae* strains and for 261 *A*. *caviae* strains isolated from faecal samples of gastroenteritis patients [33], using PRANK to perform codon aware alignment. Maximum-likelihood phylogenetic trees were constructed based on the core genome using FastTree (v 2.1.11), which uses the Jukes–Cantor model for maximum-likelihood rearrangement and the Shimodaira–Hasegawa test to generate local support values ranging from 0 to 1 by resampling the data with 1000 replicates [34,35].

### Statistical analysis

Chi-square tests were performed in R to examine the prevalence of secretion systems and toxins in *A. caviae* strains from different groups of isolation sources, and adjusted *P*-values were calculated using the Bonferroni method. Differences were considered significant at *P*<0.05.

## Results

### The complete and draft genomes of 18 *A. caviae* strains isolated from faecal samples of gastroenteritis patients

**Fig. 1.**
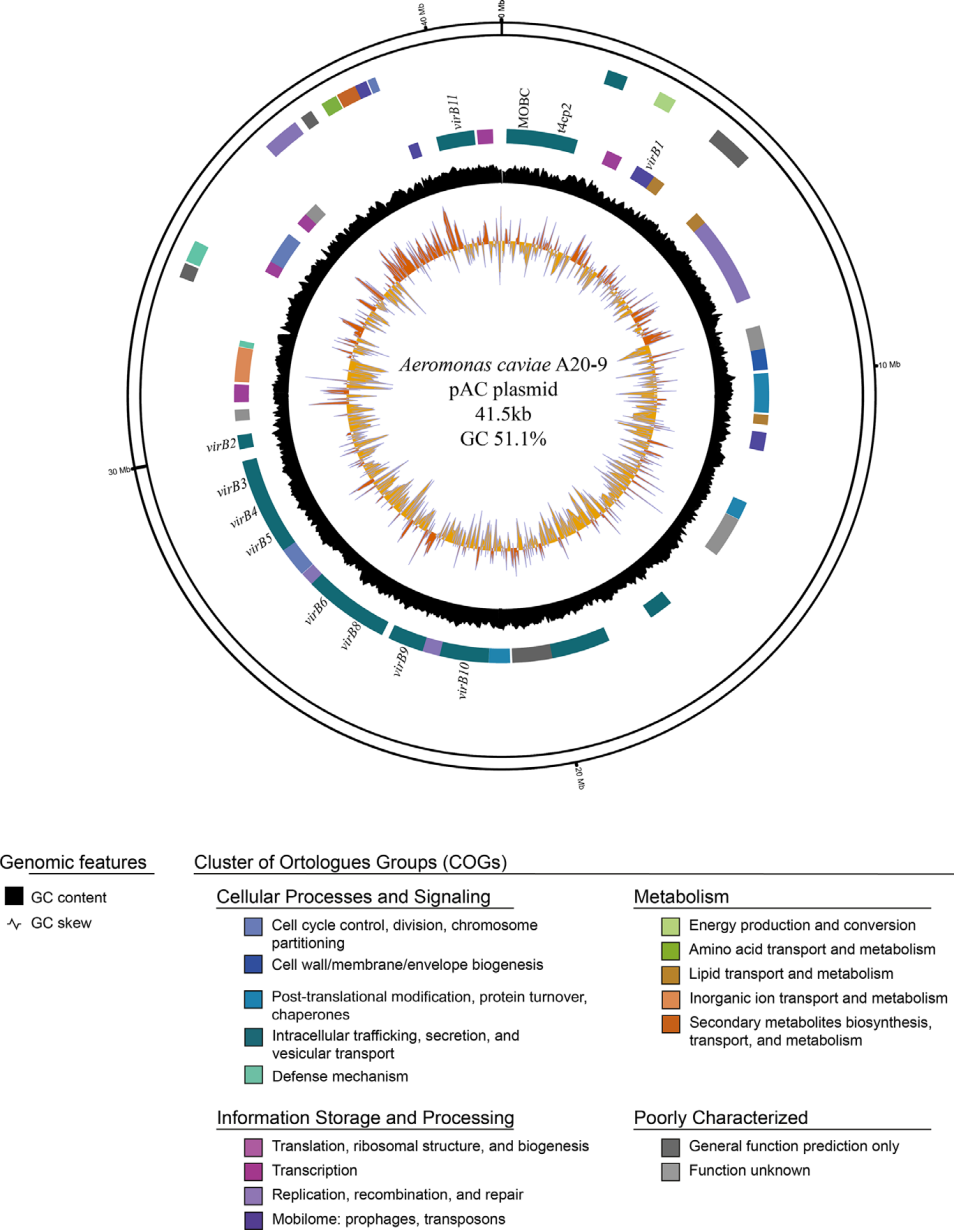
Circular representation of *A. caviae* A20-9 pAC plasmid. The pAC plasmid is 41.5 kb in size, with 50.8 % GC content and encodes 40 proteins. T4SS genes, MOBC, t4cp2, *virB1, virB2*, *virB3, virB4, virB5, virB6, virB8, virB9, virB10* and *virB11*, were annotated. Circles from inside to outside: GC skew, GC skew (orange, GC^+^; yellow, GC^-^), reverse CDS, forward CDS. Genes are colour-coded according to function. CDS: coding sequence. T4SS: type 4 secretion system.

The complete genome of *A. caviae* A20-9 was successfully obtained through hybrid assembly of the data obtained from Illumina MiSeq sequencing and Oxford Nanopore sequencing. The complete genome was 4.50 Mb in size. The chromosome was 4.46 Mb in size, with 61.9 % GC content, and encoded 4120 proteins. A circular plasmid, designated pAC, was identified in A20-9. It was 41.5 kb in length, with 50.8 % GC content, and encoded 40 proteins. The plasmid was highly similar to an unnamed plasmid in *A. hydrophila* strain Ah2111, which was isolated from a human ascites sample in China, with 94 % sequence coverage and 99 % identity. The pAC plasmid encodes T4SS proteins MOBC, t4cp2, VirB1, VirB2, VirB3, VirB4, VirB5, VirB6, VirB8, VirB8, VirB10 and VirB11 (Fig. 1).

The genomic data of the 18 *A*. *caviae* strains sequenced in this study, including the complete genome of *A. caviae* A20-9 and 17 draft genomes, are summarized in Table 1.

**Table 1.** Summary of 18 *A*. *caviae* strains sequenced and assembled in this study

Strain name	Genome assembly accession	SRA accession	Source	Level	Genome size (Mb)	GC%	No. of contigs	Plasmid	N50 (kb)	Completeness (%)	Contamination (%)
A3	JAXOHW000000000	SRR28209337	Human faeces, gastroenteritis	Draft	4.53	61.5	86	–	102.2	99.9	0
A20-3	JAXOHQ000000000	SRR28209327	Human faeces, gastroenteritis	Draft	4.56	61.5	199	–	48.8	99.9	0.29
A20-4	JAXOHR000000000	SRR28209326	Human faeces, gastroenteritis	Draft	4.48	61.5	109	–	73.4	99.9	0.29
A20-7	JAXOHS000000000	SRR28209325	Human faeces, gastroenteritis	Draft	4.29	61.8	73	–	99.9	99.9	0
A20-9	CP140118- CP140119	SRR28209331	Human faeces, gastroenteritis	Complete	4.50	61.8	2	1	–	99.9	0
A20-11	JAXOHL000000000	SRR28209340	Human faeces, gastroenteritis	Draft	4.41	61.4	88	–	87.3	100	0.58
A20-13	JAXOHM000000000	SRR28209339	Human faeces, gastroenteritis	Draft	4.55	61.4	161	–	65.6	99.9	0
A20-15	JAXOHN000000000	SRR28209330	Human faeces, gastroenteritis	Draft	4.51	61.3	168	–	54.9	99.9	0
A20-16	JAXOHO000000000	SRR28209329	Human faeces, gastroenteritis	Draft	4.45	61.8	85	–	92.5	99.9	0.58
A20-18	JAXOHP000000000	SRR28209328	Human faeces, gastroenteritis	Draft	4.60	61.3	220	–	49.7	99.9	0
A21-17	JAXOHT000000000	SRR28209324	Human faeces, gastroenteritis	Draft	4.36	62	88	–	99.4	99.9	0.29
A21-18	JAXOHU000000000	SRR28209323	Human faeces, gastroenteritis	Draft	4.53	61.6	221	–	45.3	99.9	0
A21-20	JAXOHV000000000	SRR28209338	Human faeces, gastroenteritis	Draft	4.36	62.1	77	–	110.0	99.9	0
Aero 5	JAXOIB000000000	SRR28209332	Human faeces, gastroenteritis	Draft	4.54	61.4	238	–	44.4	99.9	0
Aero11	JAXOHX000000000	SRR28209336	Human faeces, gastroenteritis	Draft	4.74	61.0	279	–	38.2	100	0.58
Aero12	JAXOHY000000000	SRR28209335	Human faeces, gastroenteritis	Draft	4.56	61.3	216	–	46.7	99.9	0.29
Aero17	JAXOHZ000000000	SRR28209334	Human faeces, gastroenteritis	Draft	4.57	61.1	241	–	42.4	99.9	0
Aero18	JAXOIA000000000	SRR28209333	Human faeces, gastroenteritis	Draft	4.51	61.3	177	–	52.6	99.9	0.29

All *A. caviae* strains, except strain A20-9, are draft assemblies. Completeness and contamination percentages were produced by the programme CheckM to assess genome quality. All strains were isolated from faecal samples of gastroenteritis patients in Sydney, Australia and sequenced in this study.

### Putative virulence factors in the complete genome of *A. caviae* A20-9 and toxins in 565 *A. caviae* strains

**Fig. 2.**
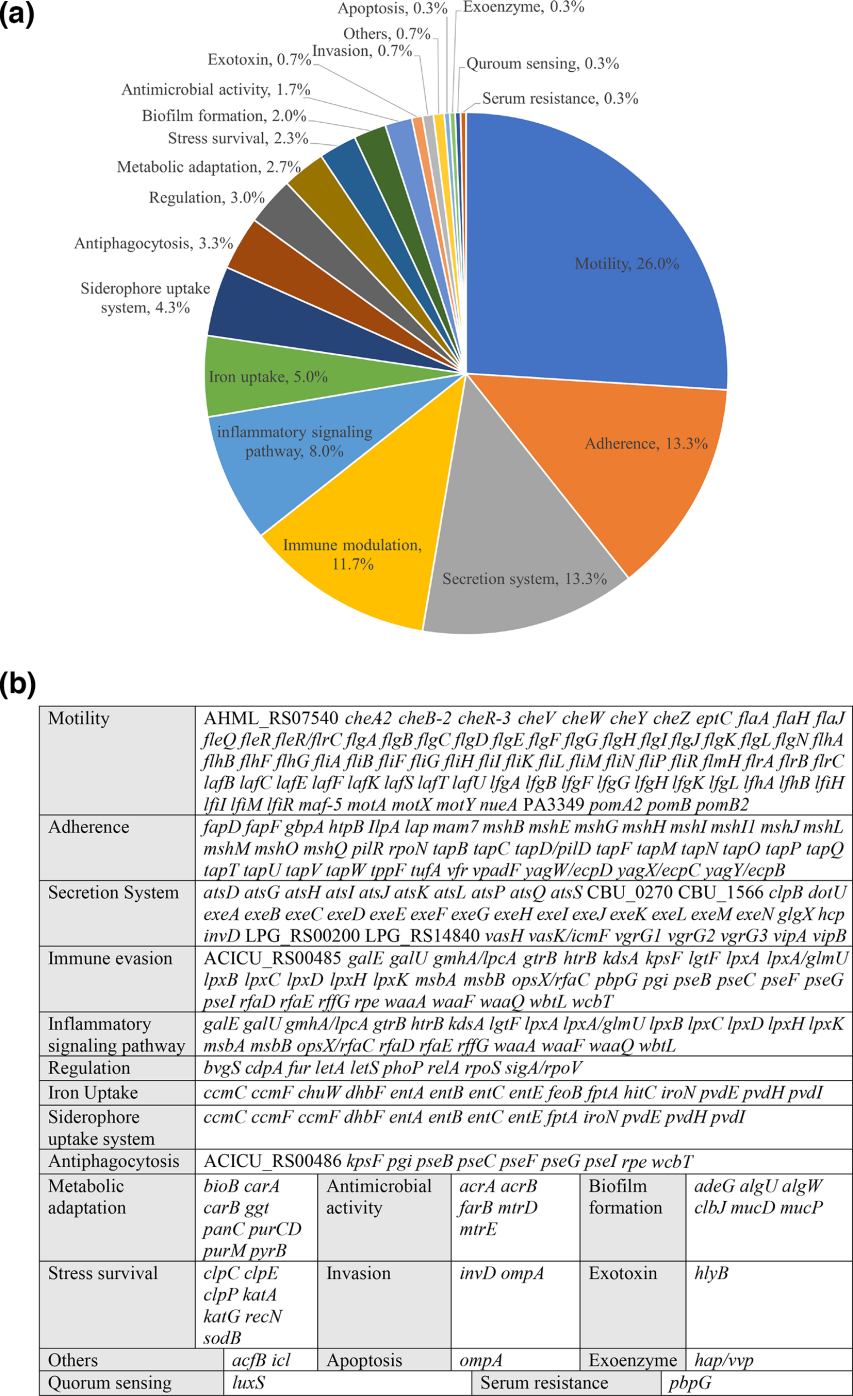
Putative virulence factors in the complete genome of *A. caviae* A20-9. Three hundred putative virulence factors were identified in the complete genome of *A. caviae* A20-9, a strain isolated from the faecal sample of a patient with gastroenteritis, through a search on the Virulence Factors Database. (**a)** Proportion of virulence factors in each category. (**b)** Virulence genes in each virulence factor category.

Three hundred putative virulence factors were identified in the complete genome of *A. caviae* A20-9 in the following categories: motility (26 %), adherence (13.3 %), secretion systems (13.3 %), immune modulation (11.7 %), inflammatory signalling pathways (8 %), iron uptake (5 %), and others (22.7 %) (Fig. 2).

The prevalence of nine toxins in 261 *A*. *caviae* strains isolated from gastroenteritis patients, 58 strains isolated from human samples other than gastroenteritis, and 246 strains from non-human samples, is summarized in Table 2. Haemolysin family protein, haemolysin III, and haemolysin D were present in all 565 strains, while thermostable haemolysin and microbial collagenase were present in 564 strains. Alt was present in 561 strains and Zot was present in 60 strains. Aerolysin and Ast were absent in all 565 *A*. *caviae* strains. No statistically significant differences were found among the groups.

**Table 2.** Prevalence of nine toxins in 565 *A*. *caviae* strains isolated from human gastroenteritis samples and other sources

Toxin	Strains from human gastroenteritis samples (%) (*n*=261)	Strains from samples of other human diseases (%) (*n*=58)	Strains from non-human samples (%) (*n*=246)
Aerolysin*	0 (0)	0 (0)	0 (0)
Thermostable cytotonic enterotoxin (Ast)†	0 (0)	0 (0)	0 (0)
Lipase (Alt)†	261 (100)	57 (98)	242 (98)
Haemolysin family protein*	261 (100)	58 (100)	246 (100)
Haemolysin III*	261 (100)	58 (100)	246 (100)
Thermostable haemolysin*	260 (99)	58 (100)	246 (100)
Haemolysin D‡	261 (100)	58 (100)	246 (100)
Microbial collagenase*	261 (100)	58 (100)	245 (99)
Zonula Occludens Toxin (Zot)*	22 (8)	8 (14)	30 (12)

Results are presented as positive strains (percentages of total strains), where *n*=the total number of strains. All 565 *A. caviae* strains analysed in this study were categorized into three groups based on isolation hosts, namely, gastroenteritis patients, humans with other diseases and non-human samples. The subscript denotes the *Aeromonas* species from which the query sequence was obtained to perform searches on blastp, where * represents *A. veronii*, † represents *A. hydrophila* and ‡ represents *A. caviae.* The prevalence of these toxins was not statistically significant among the three groups. Chi-square tests were performed to test for statistical significance.

### Prevalence of secretion systems and identification of a novel genomic island containing T4SS genes in * A. caviae*

The prevalence of secretion systems in the genomes of the 565 *A. caviae* strains was examined. Type 1 secretion system (T1SS) and type 2 secretion system (T2SS) were present in all the *A. caviae* strains, while T3SS was absent in all the *A. caviae* strains (Tables 3 and S2).

**Table 3.** Prevalence of secretion systems in 565 *A. caviae* strains isolated from human gastroenteritis samples and other sources

Secretion system	Strains from human gastroenteritis samples (%) (*n*=261)	Strains from samples of other human diseases (%) (*n*=58)	Strains from non-human samples (%) (*n*=246)
T1SS	261 (100)	58 (100)	246 (100)
T2SS	261 (100)	58 (100)	246 (100)
T3SS	0 (0)	0 (0)	0 (0)
T4SS	8 (3)	9 (16)	32 (13)
T6SS	66 (25)	9 (16)	36 (15)

Results are presented as positive strains (percentages of total strains), where *n*=the total number of strains. All 565 *A. caviae* strains analysed in this study were categorized into three groups based on isolation hosts, namely, gastroenteritis patients, humans with other diseases and non-human samples. The prevalence of T4SS and T6SS in *A. caviae* were not normally distributed across all categories (*P*<0.0001 and *P*<0.001, respectively). T4SS prevalence was significantly higher in strains from non-human samples (*P*<0.01) than in strains from human gastroenteritis samples (*P*<0.0001). T6SS prevalence was significantly higher than expected in strains from human gastroenteritis samples (*P*<0.05). Chi-square tests were performed to test for statistical significance.

**Fig. 3.**
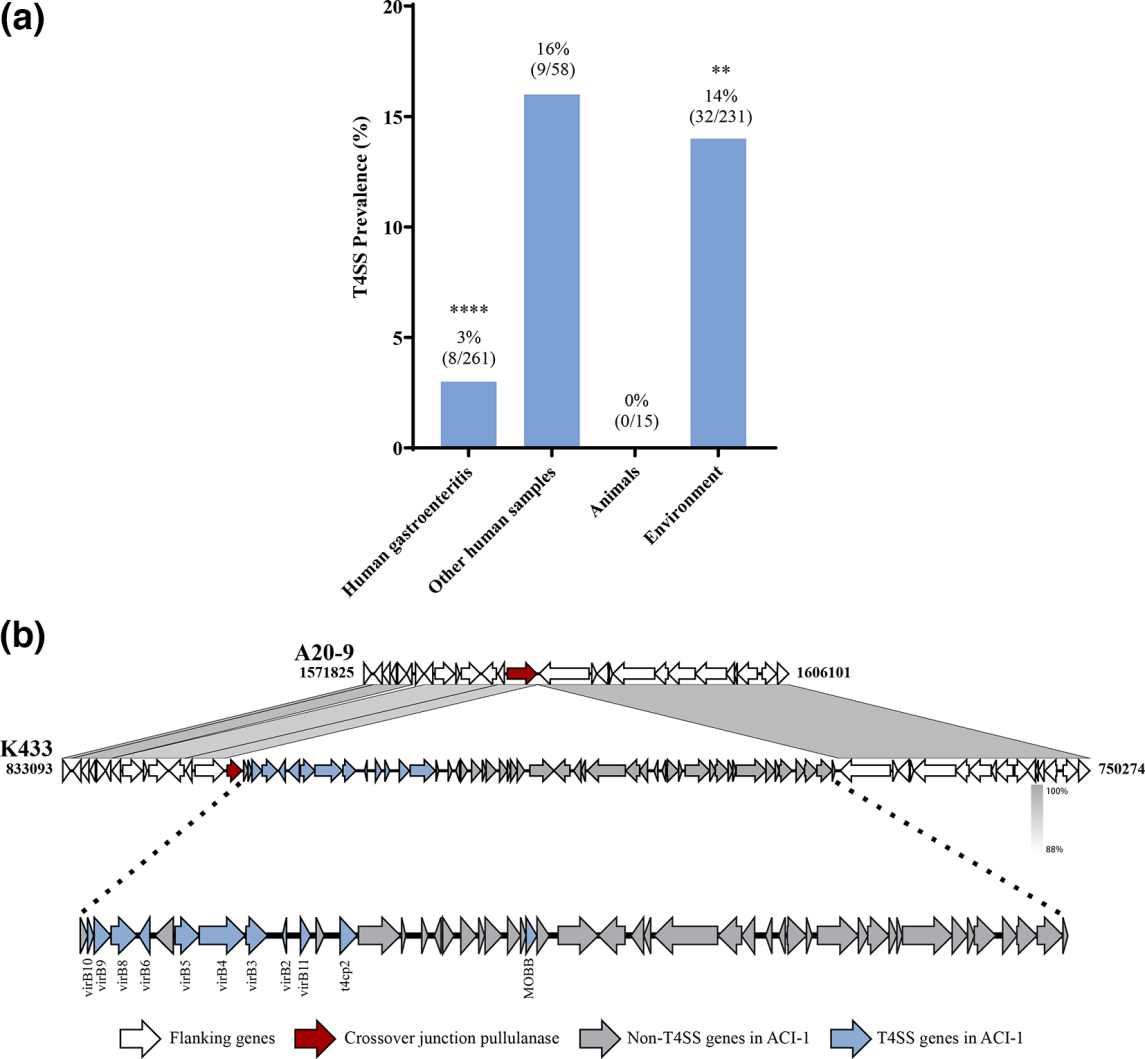
T4SS in *A. caviae* strains isolated from different sources. (a) The prevalence of T4SS in *A. caviae*. T4SS was found in 49 of the 565 (8 %) *A. caviae* strains examined in this study. The presence of T4SS in *A. caviae* was not normally distributed (*P*<0.0001). The prevalence of T4SS in *A. caviae* strains isolated from gastroenteritis patients and the environment was statistically significant (*P*<0.0001 and *P*<0.01, respectively). Statistical significance is indicated by * (**P<0.01, ****P<0.0001). Chi-square test was performed to test for statistical significance. The group ‘Animals’ consisted of strains isolated from guinea pigs, a zebu, a dog, zooplankton, freshwater fish, and mussels. The group ‘Environment’ consisted of strains isolated from the natural environment, hospital environment, farm environment, wastewater, brackish water, freshwater, and seawater.** (b)** Genes in the ACI-1 genomic island encoding T4SS components were annotated as blue arrows. The flanking genes of T4SS-positive strain K433 were compared to those in strain A20-9. The former possessed T4SS components in the chromosome, while the latter possessed T4SS components in the plasmid. T4SS: type 4 secretion system.

T4SS was present in 8 % (49/565) of the *A. caviae* strains (Table 3). The strains from non-human samples were further divided into two categories, animals and the environment, which the latter included various types of water sources. The presence of T4SS in *A. caviae* was not normally distributed across all categories (*P*<0.0001). The prevalence of T4SS was significantly lower in strains isolated from gastroenteritis patients (3 %, *P*<0.0001) than in strains isolated from environmental samples (14 %, *P*<0.01) (Fig. 3a).

We identified a novel genomic island containing T4SS genes, designated *A. caviae* genomic island-1 (ACI-1), from *A. caviae* strain K433. The ACI-1 genomic island was 49 953 bp in length and had a GC content of 62.7 %. ACI-1 is adjacent to the gene encoding a crossover junction pullulanase, a starch debranching enzyme. The components of ACI-1 are shown in Fig. 3b. Upon examination of complete genomes with T4SS, it was found to be present in either the chromosome, as in K433, or the plasmid, as in A20-9 (Fig. 1).

**Fig. 4.**
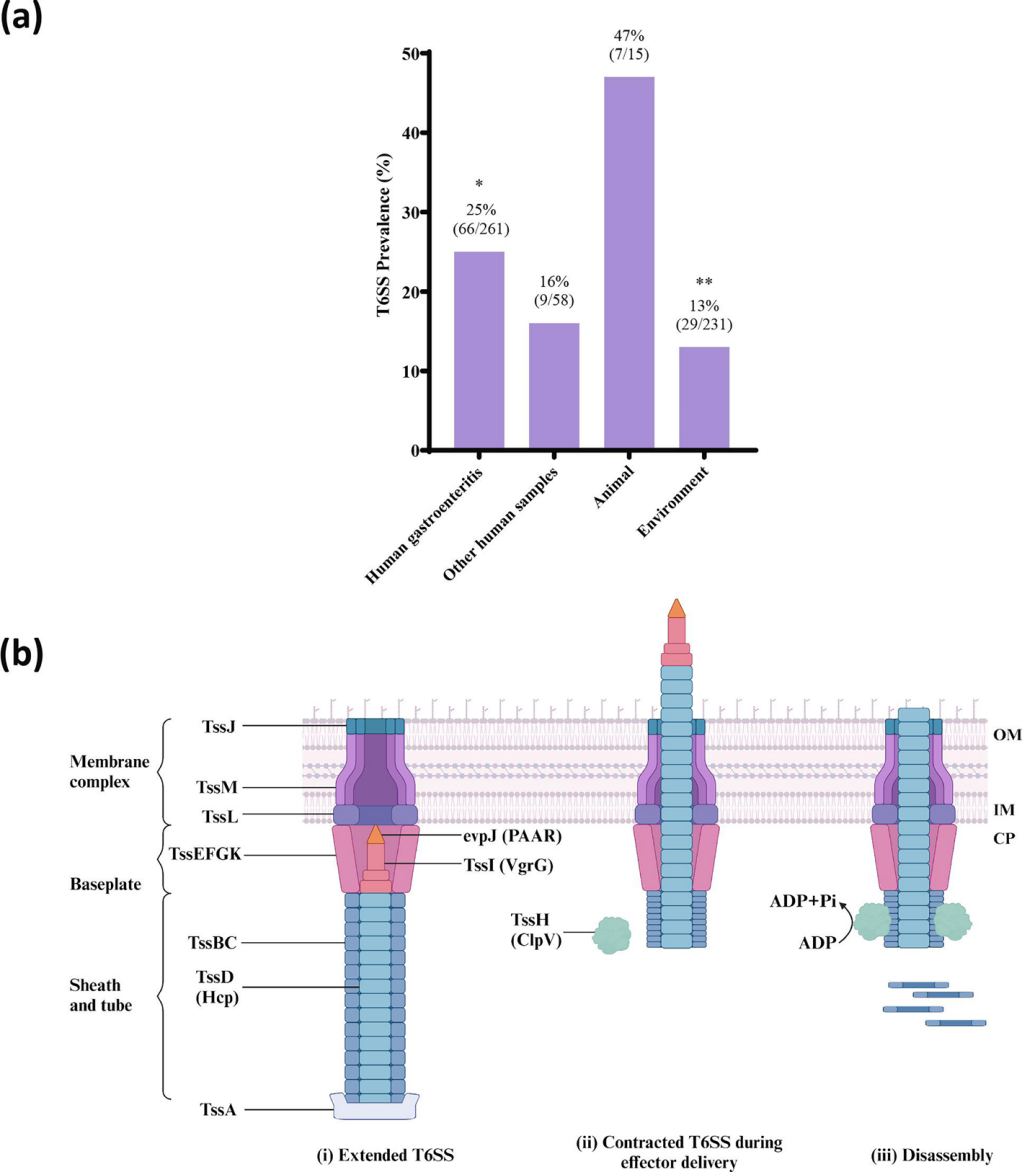
T6SS in *A. caviae* strains isolated from different sources. (a) The prevalence of the T6SS in *A. caviae.* T6SS was found in 112 of the 565 (19 %) *A. caviae* strains examined in this study. The presence of T6SS in *A. caviae* was not normally distributed (*P*<0.001). The prevalence of T6SS in *A. caviae* strains isolated from gastroenteritis patients and the environment was statistically significant (*P*<0.05 and *P*<0.01). The group ‘Animals’ consisted of strains isolated from guinea pigs, a zebu, a dog, zooplankton, freshwater fish, and mussels. The group ‘Environment’ consisted of strains isolated from the natural environment, hospital environment, farm environment, wastewater, brackish water, freshwater, and seawater. Statistical significance is indicated by * (*P<0.05, **P<0.01). Chi-square test was performed to test for statistical significance. (**b)** Schematic representation of the *A. caviae* T6SS structural and effector molecules found in this study, showing (**i**) the extended structure (ii) the contracted structure during effector delivery and (iii) disassembly of T6SS. The membrane complex is formed by proteins TssJML. The cytoplasmic baseplate is formed by TssEFGK. The sheath and tube are formed by TssBC and TssA. Effector proteins include TssD (Hcp) and TssI (VgrG). All of the labelled components are present in the T6SS encoded by *A. caviae* A20-9. OM: outer membrane. IM: inner membrane. CP: cytoplasm. T6SS: type 6 secretion system. Created with BioRender.com.

T6SS was present in 19 %(112/565) of the *A. caviae* strains (Table 3). The non-human strains were further divided into two categories, animals and the environment, which the latter included various types of water sources. The presence of T6SS in * A. caviae* was not normally distributed across all categories (*P*<0.001). The prevalence of T6SS was significantly higher in strains isolated from gastroenteritis patients (25 %, *P*<0.05) than in strains isolated from environmental samples (13 %, *P*<0.01) (Fig. 4a). The structure and effector delivery mechanism of T6SS are illustrated in Fig. 4b, where all of the labelled components are present in the T6SS encoded by *A. caviae* A20-9.

### Phylogenetic analysis of *A. caviae* genomes

**Fig. 5.**
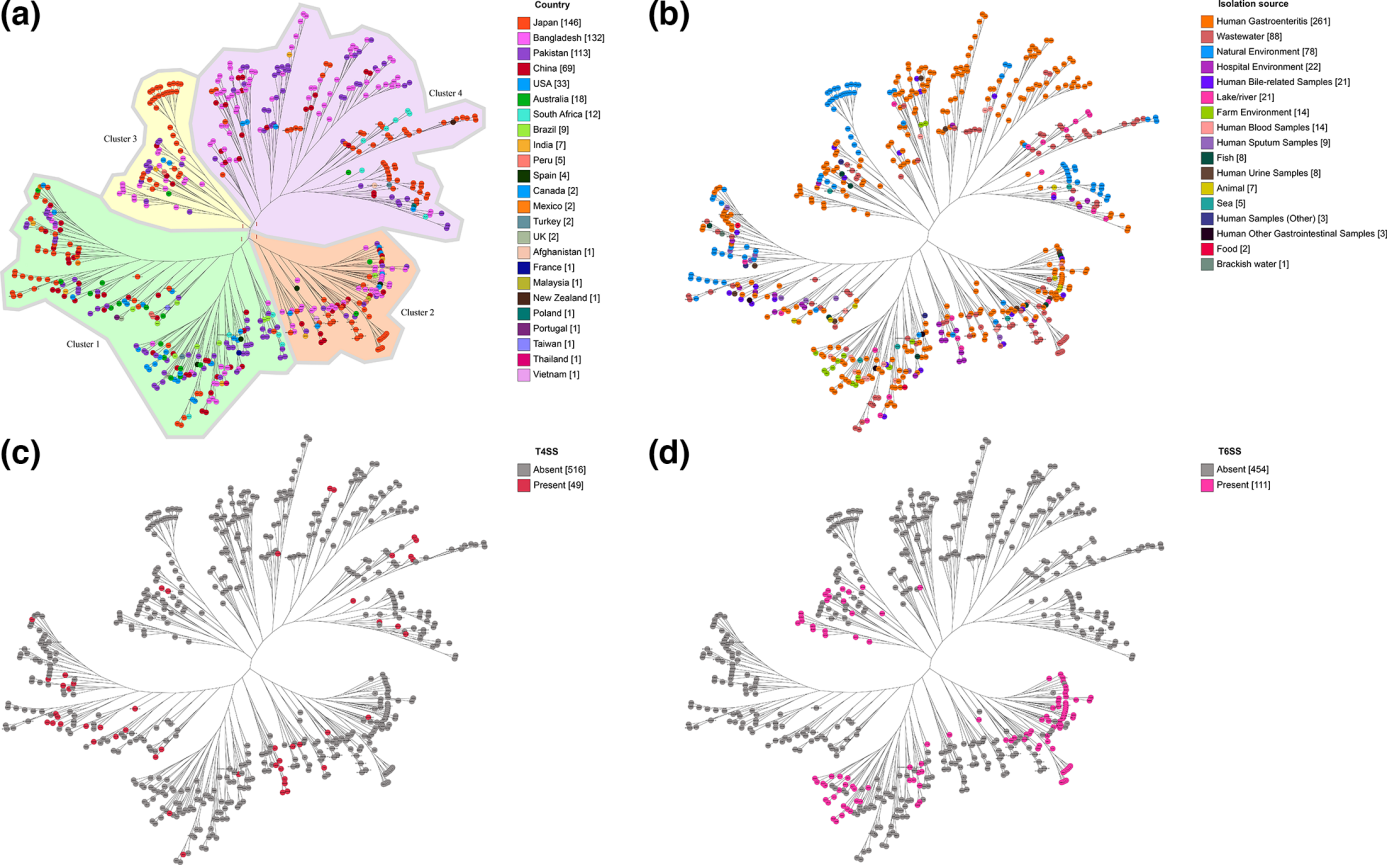
Phylogenetic tree based on the core genome of 565 *A. caviae* strains. The phylogenetic tree was generated with the maximum-likelihood method using FastTree. Strains are coloured according to (**a**) the country of isolation, (**b**) host, (**c**) presence of T4SS and (**d**) T6SS. Strains were divided into four phylogenetic clusters based on the core genome. T6SS-positive strains often formed groups in clusters 1, 2 and 3 and were nearly absent in cluster 4. Other human samples included strains isolated from a wound, a rectal swab from a healthy individual, and cerebral spinal fluid sample from a patient with subarachnoid haemorrhage. Other human gastrointestinal samples included strains isolated from intra-abdominal haematoma and abscess, and ascites. The number in brackets next to each subcategory denotes the number of strains. T4SS: type 4 secretion system. T6SS: type 6 secretion system.

Phylogenetic analysis was conducted on 565 *A. caviae* strains, as well as on 261 strains isolated from faecal samples of gastroenteritis patients. The core genome of 565 *A. caviae* strains comprised 1012 genes and formed four phylogenetic clusters (Figs 5a and S1), whereas the core genome of 261 strains isolated from faecal samples of gastroenteritis patients comprised 1546 genes and also formed four phylogenetic clusters (Figs S2 and S3). In the core-genome tree of 565 *A. caviae* strains, strains from gastroenteritis patients were evenly distributed across all clusters, representing 38–55 % of strains in each. However, Australian strains isolated from gastroenteritis patients exhibited a different distribution pattern, with 78 % (14/18) of the strains in cluster 1, while 6–11 % (1/18 or 2/18) were distributed across each of the other clusters (Fig. 5a). Notably, 85 % (6/7) of T4SS-positive strains and 75 % (3/4) of T6SS-positive strains were among the strains in cluster 1 (Fig. 5c and d), occupying different branches within the cluster. T4SS-positive strains shared the same branch as environmental strains of the same cluster, which the latter were less prevalent in the other branch containing the T6SS-positive strains. Moreover, T6SS-positive strains were nearly absent in cluster 4 and often formed groups within clusters 1, 2 and 3 (Fig. 5d).

## Discussion

In this study, we performed a comparative genomic analysis to elucidate the virulence factors and genomic features of *A. caviae* concerning its role in human gastroenteritis.

We examined the virulence factors and genes encoding toxins in 565 *A. caviae* genomes, including one complete genome and 17 draft genomes sequenced in this study (Table 1). We also identified a novel plasmid, pAC, in the complete genome of *A. caviae* A20-9 (Fig. 1). In total, 300 putative virulence factors were identified in the complete genome of *A. caviae* A20-9 (Fig. 2), indicating that *A. caviae* possesses multiple virulence factors that may contribute to the pathogenesis of gastroenteritis in humans. Toxins, including haemolysin family protein, haemolysin III, thermostable haemolysin, haemolysin D, and microbial collagenase, were present in all 565 *A. caviae* strains examined, while Alt was found in 560 (99 %) strains (Table 2). These toxins have previously been reported in other *Aeromonas* spp. [29]. As these toxins were present in nearly all of the *A. caviae* strains examined in this study, these findings suggest that they are common virulence factors in *Aeromonas* spp. Haemolysins are pore-forming toxins that lyse erythrocytes, leukocytes, and platelets [36,38]. Collagenase is involved in collagen degradation in the host extracellular matrix, and microbial collagenase is involved in the pathogenesis of *A. veronii* in infected fish [39]. Zot from *Vibrio cholerae* is involved in the degradation of zonula occludens, a component of the tight junctions between intestinal epithelial cells, whereas Zot from *Campylobacter concisus* causes intestinal epithelial cell death [40,41]. However, Zot was present in only 60 of the 565 *A. caviae* strains, 22(8 %, 22/261) of which were isolated from gastroenteritis patients. Additional studies are required to elucidate the role of these *A. caviae* toxins in the pathogenesis of human gastroenteritis. Two toxins, Ast and aerolysin, were absent in all 565 *A. caviae* strains examined in this study. The absence of aerolysin agreed with data from previous studies, which showed the absence of aerolysin in clinical *A. caviae* isolates [4,42]. Hence, aerolysin likely does not contribute to *A. caviae* pathogenesis, unlike *A. hydrophila* and *A. veronii* [30,43].

In this study, we found that T6SS had a significantly greater prevalence in *A. caviae* strains isolated from gastroenteritis patients (Fig. 4a). T6SS is involved in the virulence of some bacterial pathogens, such as *Campylobacter jejuni*, which employ secretion systems to transport virulence factors into the extracellular environment or directly into host cells [44, 45]. We identified T6SS in 19 % (112/565) of the *A. caviae* strains examined in this study (Table 3). Two effector proteins associated with T6SS, Vgr and Hcp, were identified in the complete genome of the *A. caviae* A20-9 chromosome (Fig. 2b). The VgrG1 protein in T6SS from *Aeromonas dhakensis* strain SSU was found to possess actin ADP-ribosylating activity, which induces actin cytoskeleton disruption and apoptosis in cervical cells, whereas Hcp is involved in immune modulation by evading host phagocytosis to facilitate bacterial survival in the host (Fig. 4b) [46,47]. T6SS prevalence was significantly greater in *A. caviae* strains isolated from gastroenteritis patients (25 %, *P*<0.05) than in environmental strains (13 %, *P*<0.01) (Fig. 4a). The association of T6SS positivity in human gastroenteritis strains suggests its potential role in contributing to human gastroenteritis pathogenesis. However, further investigation is needed.

T1SS and T2SS have been identified in many gram-negative bacterial species. In our study, T1SS and T2SS were found in all 565 *A. caviae* strains (Tables 3 and S2). Notably, T1SS in *A. caviae* is likely encoded by an Hly operon that consists of various *hly* genes, including *hlyD*. Haemolysin D, found in all *A. caviae* strains analysed in this study, is a homologue of the membrane fusion protein that constitutes part of the T1SS, as described in *E. coli* (Table 2) [48]. T3SS is present in many bacterial pathogens such as *Yersinia, Salmonella* and *Vibrio* species [49]. However, it was absent in *A. caviae* (Tables 3 and S2). T4SS is used by some pathogenic bacteria, for example, *Helicobacter pylori* employs T4SS to transport the virulence protein CagA into human gastric epithelial cells [50]. T4SS was identified in 8 % of the *A. caviae* strains (Table 3). T4SS prevalence was significantly lower in strains isolated from gastroenteritis patients than environmental strains (*P*<0.0001 and *P*<0.01, respectively), suggesting that T4SS may not play a major role in gastroenteritis pathogenesis in humans (Fig. 3a). Additionally, A20-9 carried T4SS in its plasmid, pAC, as opposed to the chromosome (Fig. 1). In several T4SS-positive strains, genes encoding T4SS were found on the ACI-1 genomic island (Fig. 3b), which may be potentially acquired from T4SS-carrying plasmids. The presence of pAC in *A. hydrophila* Ah2111 further suggests its mobilization potential among *Aeromonas* spp.

Based on the core genome, all 565 *A. caviae* strains formed four phylogenetic clusters, with strains isolated from gastroenteritis patients distributed across all four clusters (Fig. 5a and b). T6SS-positive strains were rare in cluster 4, while within the remaining clusters, they often formed small groups, suggesting a close phylogenetic relationship among these strains (Fig. 5d). The predominance of Australian strains in cluster 1 (78 %, 14/18) (Fig. 5a) further supports evidence from a previous study, which demonstrated that most gastroenteritis patients (96 %) acquired *Aeromonas* enteric infections locally in Australia, with no history of overseas travel [2].

In summary, we identified novel genomic features of *A. caviae* including a plasmid and a genomic island encoding T4SS. We showed that aerolysin, Ast and T3SS were absent in *A. caviae*. We discovered that T6SS-carrying *A. caviae* strains were more often found in gastroenteritis patients. Furthermore, we showed that *A. caviae* formed four phylogenetic clusters based on the core genome and that T6SS-carrying *A. caviae* strains were often phylogenetically closely related. Our findings reveal novel genomic features of *A. caviae* and suggest that T6SS may play a role in human gastroenteritis.

## supplementary material

10.1099/mgen.0.001258 Uncited Supplementary Material 1.

10.1099/mgen.0.001258 Uncited Supplementary Material 2.

## Abbreviations

ACI-1: *A. caviae* genomic island-1
Alt: lipase
Ast: thermostable cytotonic enterotoxin
IBD: inflammatory bowel disease
NCBI: National Center for Biotechnology Information
RAST: rapid annotation using subsystem technology
SRA: sequence read archive
T1SS: type 1 secretion system
T2SS: type 2 secretion system
T3SS: type 3 secretion System
T4SS: type 4 secretion system
T6SS: type 6 secretion system
VFDB: virulence factors database
Zot: zonula occludens toxin
