# Supplementary material for: Analysis of global Aeromonas caviae genomes revealed that strains carrying T6SS are more common in human gastroenteritis than in environmental sources and are often phylogenetically related
Source: Microb Genom. 2024 May 30;10(5):001258. doi: 10.1099/mgen.0.001258 (PMC11165597; doi:10.1099/mgen.0.001258)
Supplement: Uncited Supplementary Material 1. [file mgen-10-01258-s001.pdf]

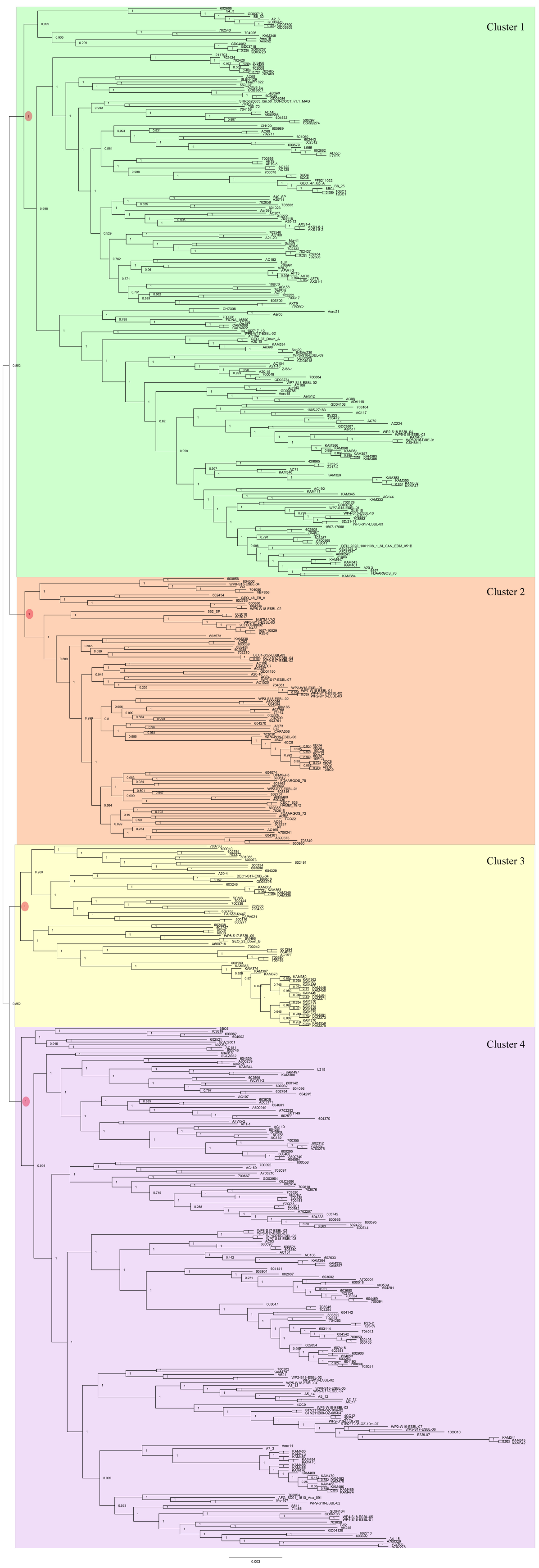

Figure S1. Rectangular phylogenetic tree based on the core genome of 565 *A. caviae* strains. Four phylogenetic clusters were identified in the core genome phylogenetic tree of 565 *A. caviae* strains. This was determined by local support values of 1 in the main branches, as highlighted in red.

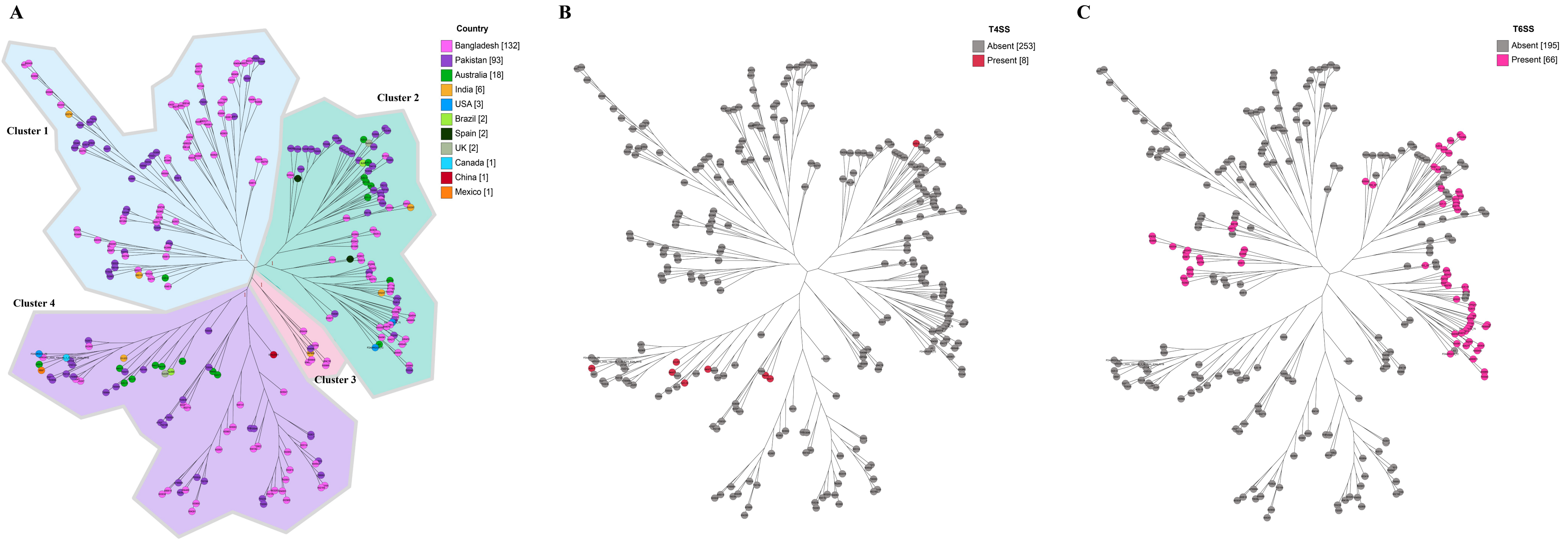

Figure S2. Phylogenetic tree based on the core genome of 261 *A. caviae* strains isolated from faecal samples of gastroenteritis patients. The phylogenetic tree was generated with the maximum likelihood method using FastTree. Strains are coloured according to the country of isolation **(A)**, presence of T4SS **(B)** and T6SS **(C)**. Strains were divided into four phylogenetic clusters based on the core genome.

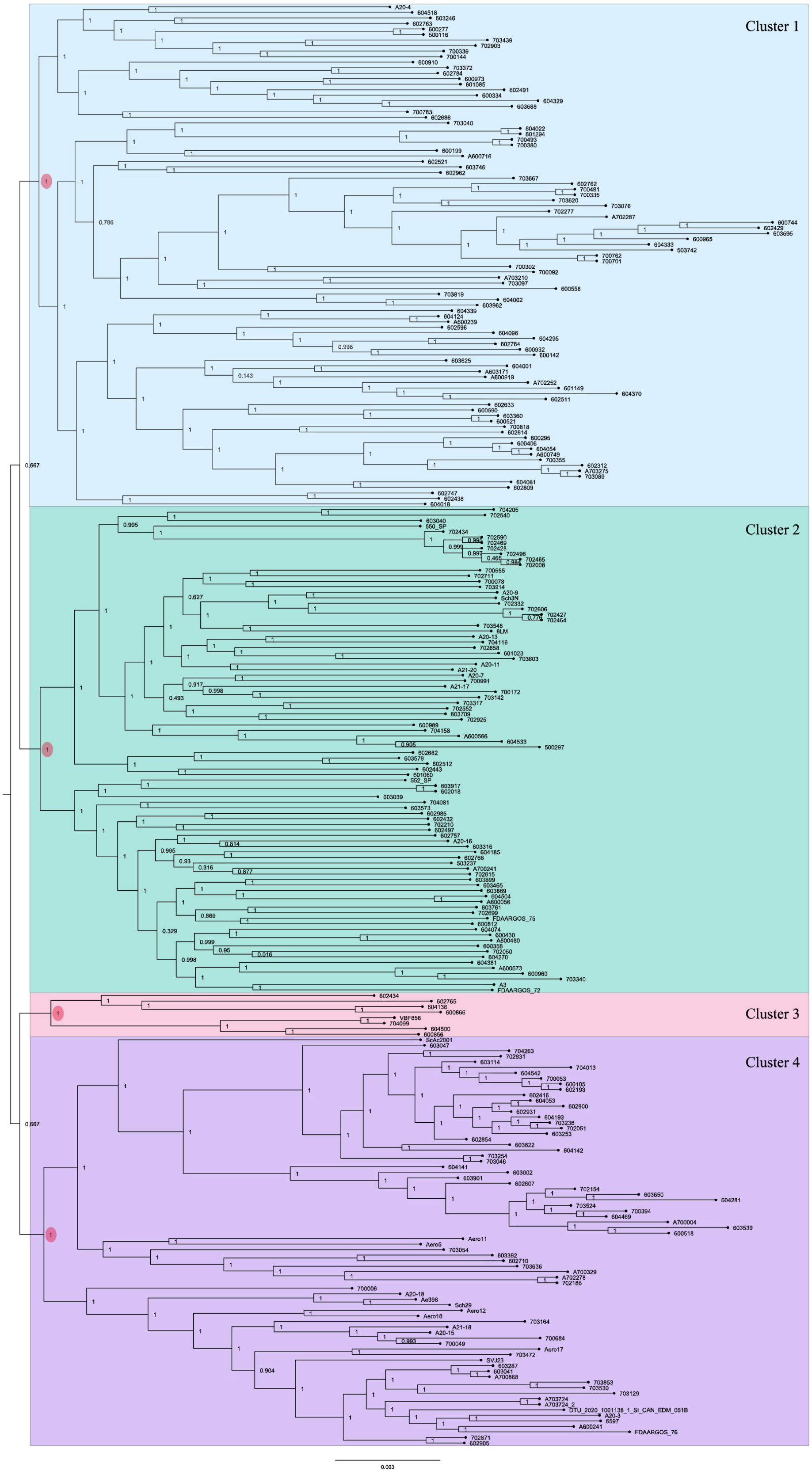

Figure S3. Rectangular phylogenetic tree based on the core genome of 261 *A. caviae* strains isolated from faecal samples of gastroenteritis patients. Four phylogenetic clusters were identified in the core genome phylogenetic tree of 261 *A. caviae* strains isolated from faecal samples of gastroenteritis patients. Each cluster is coloured by a different colour. This was determined by local support values of 1 in the main branches, as highlighted in red.

Table S1. Summary of *A. caviae* strains retrieved from the NCBI Genome and SRA databases.

NCBI RefSeq / SRA Accession ID	Strain names	Country	Source	Level	Size (Mb)	GC (%)	N50 (kb)	No. of contigs	Plasmid	Completeness (%)	Contamination (%)	Reference
GCA_019972735.1	KAM329	Japan	Aquatic animals, plants, environment	Draft	4.91	60.8	36.5	371	-	100.0	0.58	
GCA_019972535.1	KAM333	Japan	Aquatic animals, plants, environment	Draft	4.57	61.2	47.4	472	-	99.9	0.00	
GCA_019972515.1	KAM334	Japan	Aquatic animals, plants, environment	Draft	4.55	61.3	66.7	214	-	99.9	0.29	
GCA_019972495.1	KAM335	Japan	Aquatic animals, plants, environment	Draft	4.58	61.3	58.2	318	-	99.9	0.00	
GCA_019972475.1	KAM336	Japan	Aquatic animals, plants, environment	Draft	4.89	60.7	53.0	426	-	99.9	0.29	
GCA_019972455.1	KAM337	Japan	Aquatic animals, plants, environment	Draft	4.76	61.0	56.3	313	-	100.0	0.00	
GCA_019973835.2	KAM339	Japan	Aquatic animals, plants, environment	Complete	5.01	61.3	-	8	7	99.9	0.00	
GCA_019971135.1	KAM340	Japan	Aquatic animals, plants, environment	Draft	4.88	60.7	51.9	371	-	99.9	0.29	
GCA_019971155.1	KAM341	Japan	Aquatic animals, plants, environment	Draft	4.89	60.4	33.7	526	-	100.0	0.00	
GCA_019971175.1	KAM342	Japan	Aquatic animals, plants, environment	Draft	4.92	60.4	33.5	536	-	100.0	0.00	
GCA_019971195.1	KAM343	Japan	Aquatic animals, plants, environment	Draft	4.97	60.5	34.7	562	-	100.0	0.58	
GCA_022835735.1	KAM344	Japan	Aquatic animals, plants, environment	Draft	4.63	61.1	31.6	515	-	99.9	0.00	
GCA_019972775.2	KAM345	Japan	Aquatic animals, plants, environment	Complete	4.85	61.2	-	7	6	99.9	0.00	
GCA_019971215.1	KAM346	Japan	Aquatic animals, plants, environment	Draft	4.89	61.1	51.7	390	-	100.0	0.58	
GCA_019971305.1	KAM347	Japan	Aquatic animals, plants, environment	Draft	4.81	60.9	37.0	527	-	100.0	0.00	
GCA_019971385.1	KAM348	Japan	Aquatic animals, plants, environment	Draft	4.81	61.3	76.0	294	-	99.9	0.58	
GCA_019971485.1	KAM350	Japan	Aquatic animals, plants, environment	Draft	4.80	60.9	36.9	437	-	100.0	0.00	

GCA_019971515.1	KAM351	Japan	Aquatic animals, plants, environment	Draft	4.97	60.7	49.9	320	-	99.9	0.29
GCA_019971575.1	KAM352	Japan	Aquatic animals, plants, environment	Draft	4.76	60.9	41.9	394	-	100.0	0.00
GCA_019971595.1	KAM353	Japan	Aquatic animals, plants, environment	Draft	4.83	61.0	45.1	451	-	99.9	0.29
GCA_019971635.1	KAM355	Japan	Aquatic animals, plants, environment	Draft	4.83	61.2	68.8	282	-	99.9	0.58
GCA_019971655.1	KAM356	Japan	Aquatic animals, plants, environment	Draft	4.54	61.4	48.6	323	-	99.9	0.00
GCA_019971675.1	KAM357	Japan	Aquatic animals, plants, environment	Draft	4.53	61.5	48.2	276	-	99.9	0.00
GCA_019971715.1	KAM359	Japan	Aquatic animals, plants, environment	Draft	4.79	61.3	75.6	181	-	100.0	1.17
GCA_019971735.1	KAM360	Japan	Aquatic animals, plants, environment	Draft	4.75	60.9	45.1	531	-	99.9	0.00
GCA_019971755.1	KAM361	Japan	Aquatic animals, plants, environment	Draft	4.53	61.5	46.5	299	-	99.9	0.00
GCA_019971775.1	KAM362	Japan	Aquatic animals, plants, environment	Draft	4.86	61.2	68.3	251	-	99.9	0.58
GCA_019971795.1	KAM363	Japan	Aquatic animals, plants, environment	Draft	4.52	61.5	44.0	284	-	99.9	0.00
GCA_019971815.1	KAM364	Japan	Aquatic animals, plants, environment	Draft	4.58	61.3	48.9	343	-	99.9	0.00
GCA_019971835.1	KAM365	Japan	Aquatic animals, plants, environment	Draft	4.86	61.2	63.2	250	-	99.9	0.58
GCA_019971855.1	KAM366	Japan	Aquatic animals, plants, environment	Draft	4.52	61.5	45.7	287	-	99.9	0.00
GCA_019971875.1	KAM367	Japan	Aquatic animals, plants, environment	Draft	4.86	61.2	68.3	251	-	99.9	0.58
GCA_019971895.1	KAM368	Japan	Aquatic animals, plants, environment	Draft	4.52	61.5	45.0	290	-	99.9	0.00
GCA_019971915.1	KAM369	Japan	Aquatic animals, plants, environment	Draft	4.84	61.3	91.4	172	-	100.0	1.17
GCA_019971935.1	KAM370	Japan	Aquatic animals, plants, environment	Draft	4.79	61.3	90.6	150	-	100.0	1.17
GCA_022835755.1	KAM371	Japan	Aquatic animals, plants, environment	Draft	4.82	61.2	71.5	218	-	99.9	0.58

GCA_019971955.1	KAM372	Japan	Aquatic animals, plants, environment	Draft	4.78	61.3	104.3	153	-	100.0	1.17
GCA_019971975.1	KAM373	Japan	Aquatic animals, plants, environment	Draft	4.78	61.3	83.8	162	-	100.0	1.17
GCA_019971995.1	KAM374	Japan	Aquatic animals, plants, environment	Draft	4.79	61.2	71.8	217	-	99.9	0.58
GCA_019972015.1	KAM375	Japan	Aquatic animals, plants, environment	Draft	4.83	61.3	71.2	166	-	100.0	1.17
GCA_019972755.1	KAM376	Japan	Aquatic animals, plants, environment	Draft	4.84	61.4	101.0	176	-	100.0	1.17
GCA_019972715.1	KAM377	Japan	Aquatic animals, plants, environment	Draft	4.81	61.4	107.0	148	-	100.0	1.17
GCA_019972035.1	KAM378	Japan	Aquatic animals, plants, environment	Draft	4.82	61.2	65.6	235	-	99.9	0.58
GCA_019972575.1	KAM379	Japan	Aquatic animals, plants, environment	Draft	4.89	61.3	94.0	163	-	100.0	1.17
GCA_019972615.1	KAM381	Japan	Aquatic animals, plants, environment	Draft	4.78	61.3	89.1	157	-	100.0	1.17
GCA_019972635.1	KAM382	Japan	Aquatic animals, plants, environment	Draft	4.82	61.2	73.9	234	-	99.9	0.58
GCA_019972655.1	KAM383	Japan	Aquatic animals, plants, environment	Draft	4.72	61.0	38.6	373	-	100.0	0.00
GCA_019972675.1	KAM384	Japan	Aquatic animals, plants, environment	Draft	4.65	61.3	49.0	291	-	99.9	0.88
GCA_022835775.1	KAM447	Japan	Aquatic animals, plants, environment	Draft	4.67	61.2	40.0	355	-	99.9	0.00
GCA_022835795.1	KAM448	Japan	Aquatic animals, plants, environment	Draft	4.85	61.2	48.5	245	-	99.9	0.58
GCA_022835815.1	KAM449	Japan	Aquatic animals, plants, environment	Draft	4.82	61.2	57.9	217	-	100.0	0.58
GCA_022835835.1	KAM450	Japan	Aquatic animals, plants, environment	Draft	4.81	61.3	53.3	240	-	99.9	0.58
GCA_022835855.1	KAM451	Japan	Aquatic animals, plants, environment	Draft	4.81	61.2	57.8	214	-	99.9	0.58
GCA_022835915.1	KAM463	Japan	Aquatic animals, plants, environment	Draft	4.46	61.5	28.1	412	-	99.9	0.58
GCA_022835935.1	KAM465	Japan	Aquatic animals, plants, environment	Draft	4.69	60.9	29.8	474	-	99.9	0.58

GCA_022835955.1	KAM466	Japan	Aquatic animals, plants, environment	Draft	4.68	60.9	30.9	478	-	100.0	0.58
GCA_022835975.1	KAM467	Japan	Aquatic animals, plants, environment	Draft	4.75	60.9	25.6	543	-	100.0	0.58
GCA_022835995.1	KAM468	Japan	Aquatic animals, plants, environment	Draft	4.69	60.9	33.1	470	-	100.0	0.58
GCA_022836015.1	KAM469	Japan	Aquatic animals, plants, environment	Draft	4.77	60.9	28.4	476	-	100.0	0.58
GCA_022836035.1	KAM470	Japan	Aquatic animals, plants, environment	Draft	4.72	60.9	31.1	499	-	100.0	0.58
GCA_024347695.1	KAM471	Japan	Aquatic animals, plants, environment	Complete	4.69	61.3	-	5	4	99.9	0.00
GCA_022836095.1	KAM473	Japan	Aquatic animals, plants, environment	Draft	4.51	61.2	31.7	467	-	99.9	0.00
GCA_022836115.1	KAM474	Japan	Aquatic animals, plants, environment	Draft	4.69	60.9	30.9	488	-	100.0	0.58
GCA_022836135.1	KAM475	Japan	Aquatic animals, plants, environment	Draft	4.72	61.0	25.6	497	-	99.9	0.58
GCA_022836155.1	KAM476	Japan	Aquatic animals, plants, environment	Draft	4.68	60.9	24.0	528	-	99.9	0.00
GCA_022836195.1	KAM478	Japan	Aquatic animals, plants, environment	Draft	4.73	60.9	33.3	490	-	100.0	0.58
GCA_026000055.1	KAM479	Japan	Aquatic animals, plants, environment	Complete	4.99	60.9	-	6	5	100.0	0.00
GCA_022836235.1	KAM480	Japan	Aquatic animals, plants, environment	Draft	4.72	60.8	25.5	575	-	99.9	0.58
GCA_022836255.1	KAM481	Japan	Aquatic animals, plants, environment	Draft	4.63	61.3	35.7	349	-	99.9	0.29
GCA_022836275.1	KAM482	Japan	Aquatic animals, plants, environment	Draft	4.72	60.9	29.1	494	-	100.0	0.58
GCA_022836295.1	KAM483	Japan	Aquatic animals, plants, environment	Draft	4.52	61.2	29.5	470	-	99.9	0.00
GCA_022836315.1	KAM484	Japan	Aquatic animals, plants, environment	Draft	4.71	61.0	31.0	477	-	99.9	0.58
GCA_022836335.1	KAM485	Japan	Aquatic animals, plants, environment	Draft	4.69	60.9	31.5	473	-	100.0	0.58
GCA_022836355.1	KAM486	Japan	Aquatic animals, plants, environment	Draft	4.80	61.3	50.6	230	-	99.9	0.58

GCA_024347715.1	KAM497	Japan	Aquatic animals, plants, environment	Complete	4.88	61.1	-	7	6	99.9	0.00	
GCA_024347775.1	KAM643	Japan	Aquatic animals, plants, environment	Complete	4.86	61.2	-	4	3	99.9	0.29	
GCA_021654375.1	NUITM-VA2	Vietnam	Aquatic animals, plants, environment	Complete	5.04	61.1	-	1	-	99.9	0.00	
GCA_026309375.1	CAPA006	Peru	*Arapaima gigas* kidney/liver, hemorrhagic disease	Draft	4.59	61.3	201.1	68	-	99.9	0.00	
GCA_026309435.1	CAPA007	Peru	*Arapaima gigas* kidney/liver, hemorrhagic disease	Draft	4.52	61.7	329.4	45	-	99.9	0.00	
GCA_012102435.1	UFMG-H8	Brazil	*Bos indicus* urine	Draft	4.55	61.7	410.8	33	-	99.9	0.00	[1]
GCA_003322775.1	AFG_SD01_1510_Aca_091	Afghanistan	*Canis lupus familiaris* faeces	Draft	4.50	61.1	68.9	228	-	99.6	1.17	[2]
GCA_018360005.1	CECT 838	Spain	*Cavia porcellus* epizootic spleen	Draft	4.48	61.8	287.2	51	-	99.9	0.00	
GCA_001183595.1	A23	China	Chicken from supermarket	Draft	4.52	61.3	64.4	219	-	99.9	0.00	[3]
GCA_026309475.1	CAPA021	Peru	*Colossoma macropomum* kidney/liver hemorrhagic disease	Draft	4.40	61.7	259.9	50	-	99.9	0.00	
GCA_016728865.1	Aero19	Brazil	Combined sewer	Complete	4.48	61.5	-	2	1	99.9	0.58	
GCA_016126815.2	Aero21	Brazil	Combined sewer	Complete	5.37	60.5	-	2	1	99.4	0.00	
GCA_016598815.1	Aero52	Brazil	Combined sewer	Complete	4.53	61.4	-	4	3	99.9	0.58	
GCA_028767035.1	GLB-10	China	Estuary water	Draft	4.42	61.7	69.2	167	-	99.9	0.00	
GCA_019711295.1	Colony274	Thailand	Food	Draft	4.68	62.4	34.9	436	-	96.4	0.00	
GCA_000813475.1	L12	Malaysia	Freshwater lake	Draft	4.38	61.7	126.3	91	-	99.9	0.58	[4]
GCA_003350165.1	HAMBI 1972	USA	Guinea Pig	Draft	4.46	61.8	154.4	61	-	99.9	0.00	[5]
GCA_029844445.1	GD03687	Pakistan	Hospital sink basin	Draft	5.08	60.7	4822.8	2	-	100.0	0.00	
GCA_029843805.1	GD03707	Pakistan	Hospital sink basin	Draft	4.85	60.9	4639.1	2	-	99.9	0.00	
GCA_029842335.1	GD03784	Pakistan	Hospital sink basin	Draft	4.73	61.1	89.4	145	-	100.0	0.44	
GCA_029842185.1	GD03796	Pakistan	Hospital sink basin	Draft	5.14	60.6	4666.0	12	-	100.0	0.44	
GCA_029841915.1	GD03805	Pakistan	Hospital sink basin	Draft	5.07	60.6	50.2	249	-	99.9	0.00	

GCA_029836355.1	GD04082	Pakistan	Hospital sink basin	Draft	4.96	61.0	4733.6	2	-	99.9	0.44	
GCA_029835835.1	GD04108	Pakistan	Hospital sink basin	Draft	4.84	60.9	4676.9	2	-	99.9	0.15	
GCA_029835355.1	GD04134	Pakistan	Hospital sink basin	Draft	4.34	61.7	49.9	189	-	99.9	0.00	
GCA_029834995.1	GD04150	Pakistan	Hospital sink basin	Draft	4.33	62.1	399.4	28	-	98.7	0.00	
GCA_029843795.1	GD03710	Pakistan	Hospital sink drain	Draft	5.09	60.9	39.7	310	-	99.9	1.02	
GCA_029843365.1	GD03732	Pakistan	Hospital sink drain	Draft	4.66	61.1	68.5	191	-	99.9	0.15	
GCA_029842325.1	GD03788	Pakistan	Hospital sink drain	Draft	4.96	60.7	4700.0	4	-	99.9	0.44	
GCA_029839565.1	GD03928	Pakistan	Hospital sink drain	Draft	5.34	60.5	5086.3	7	-	99.9	0.00	
GCA_029839065.1	GD03954	Pakistan	Hospital sink drain	Draft	4.30	61.7	66.1	155	-	99.3	0.58	
GCA_029838445.1	GD03989	United States	Hospital sink drain	Draft	4.52	61.2	55.2	207	-	99.9	0.29	
GCA_029837645.1	GD04018	United States	Hospital sink drain	Draft	4.52	61.2	58.9	208	-	99.9	0.29	
GCA_029836265.1	GD04086	Pakistan	Hospital sink drain	Draft	4.81	61.1	4574.5	5	-	99.9	0.44	
GCA_029843955.1	GD03700	Pakistan	Hospital sink faucet	Draft	5.38	60.4	5105.3	7	-	99.9	0.00	
GCA_029843685.1	GD03718	Pakistan	Hospital sink faucet	Draft	4.55	61.3	48.9	222	-	99.9	0.00	
GCA_029843565.1	GD03720	Pakistan	Hospital sink faucet	Draft	4.57	61.3	60.6	202	-	99.9	0.00	
GCA_029835935.1	GD04103	Pakistan	Hospital sink faucet	Draft	4.48	61.6	45.2	205	-	99.9	0.00	
GCA_029835415.1	GD04129	Pakistan	Hospital sink faucet	Draft	4.62	61.4	53.0	216	-	99.9	0.44	
GCA_024218755.1	FAHZZU2447	China	Human bile, biliary tract infection	Complete	4.79	61.1	-	3	2	99.9	0.44	[6]
GCA_020405325.1	71442	China	Human bile, cholecystitis	Complete	4.44	61.7	-	1	-	99.9	0.00	
GCA_020640975.1	71485	China	Human bile, cholecystitis	Complete	4.61	61.2	-	2	1	99.9	0.00	
GCA_013487985.1	1507-17068	China	human bile, obstructive jaundice	Complete	4.53	61.3	-	1	-	99.9	0.58	
GCA_022343945.1	211703	China	Human cerebrospinal fluid, cerebral infection	Complete	4.78	61.8	-	1	-	99.8	0.00	[7]
GCA_023093415.1	6597	Mexico	Human faeces, diarrhea	Draft	4.46	61.4	93.5	167	-	99.9	0.00	[8]

GCA_000959705.2	8LM	Brazil	Human faeces, diarrhea	Complete	4.55	61.7	-	1	-	99.3	0.00	[9]
GCA_025565345.1	A703724	Pakistan	Human faeces, diarrhea	Draft	4.33	61.8	107.0	89	-	99.9	0.00	
GCA_000783775.2	FDAARGOS_72	USA	Human faeces, diarrhea	Complete	4.52	61.7	-	1	-	99.9	0.00	[10]
GCA_000783715.2	FDAARGOS_75	USA	Human faeces, diarrhea	Draft	4.56	61.7	4555.4	1	-	99.9	0.00	[10]
GCA_000783695.2	FDAARGOS_76	USA	Human faeces, diarrhea	Draft	4.88	61.0	3359.5	10	-	99.9	0.00	[10]
GCA_009831085.1	ScAc2001	China	Human faeces, diarrhea	Draft	4.49	61.1	27.3	340	-	98.2	0.00	[11]
GCA_018359875.1	550_SP	Spain	Human faeces, gastroenteritis	Draft	4.52	61.7	393.7	47	-	99.9	0.00	
GCA_018359805.1	552_SP	Spain	Human faeces, gastroenteritis	Draft	4.46	61.7	392.1	41	-	99.9	0.00	
GCA_901202955.1	Sch29	UK	Human faeces, gastroenteritis	Draft	4.42	61.3	86.9	177	-	99.9	0.29	[12]
GCA_901212305.1	Sch3N	UK	Human faeces, gastroenteritis	Draft	4.73	61.4	90.4	169	-	99.9	0.29	[12]
GCA_026013215.1	SVJ23	India	Human faeces, gastroenteritis	Draft	4.56	61.5	76.8	182	-	100.0	0.00	
GCA_001702475.1	VBF856	India	Human faeces, gastroenteritis	Draft	4.50	61.4	75.1	216	-	98.7	0.00	[13]
GCA_000208825.1	Ae398	Brazil	Human faeces, gastroenteritis and diarrhea	Draft	4.44	61.4	76.4	149	-	99.9	0.00	
GCA_009906335.1	ADV118	USA	Human hematoma, intra-abdominal	Draft	4.39	61.5	60.4	149	-	99.9	0.00	[14]
GCA_018359825.1	549_SP	Spain	Human intra-abdominal abscess, abscessus	Draft	4.50	61.2	223.1	79	-	99.9	0.00	[15]
GCA_029773025.1	FIONA 16800	Taiwan	Human plasma	Complete	5.32	60.4	-	3	2	100.0	0.73	
GCA_900491665.1	ZJ33-3	China	Human rectal swab	Draft	4.48	61.5	59.7	223	-	100.0	0.00	[16]
GCA_013487965.1	1607-10029	China	Human shunt fluid, cholangitis	Complete	4.52	61.6	4520.7	1	-	99.3	0.00	

GCA_013488005.1	1605-27183	China	Human shunt fluid, postoperative cholangiocarcinoma	Complete	4.86	61.0	-	1	-	99.9	0.58	
GCA_023921525.1	21006	China	Human sputum, lymphadenoma	Complete	4.57	61.4	-	1	-	99.9	0.29	
GCA_020181575.1	K433	China	Human sputum, pneumonia	Complete	4.68	61.4	-	1	-	99.9	0.00	[17]
GCA_029590475.1	AC1520	China	Human urine	Complete	4.74	61.1	-	2	1	99.9	0.08	
GCA_001270765.1	429865	Mexico	Human urine, with cytotoxic activity	Draft	4.70	61.0	1256.3	4	-	99.3	0.00	[18]
GCA_004024495.1	BVH98	USA	Human wound	Draft	4.53	61.7	37.9	253	-	99.9	0.00	[14]
GCA_025755795.1	SD/21-11	India	*Labeo rohita*, fresh water pond	Draft	4.24	62.0	101.7	99	-	99.9	0.00	[19]
GCA_009906325.1	AK245	USA	Lake water	Draft	4.45	61.6	48.9	212	-	99.9	0.00	[14]
GCA_029223215.1	SCMS	China	*Micropterus salmoides*	Draft	4.33	61.7	26.1	306	-	99.2	0.05	
GCA_026797975.1	Mu-167	Turkey	Mucilage in the Sea of Marmara	Draft	4.30	61.7	50.8	186	-	99.3	0.00	
GCA_026797935.1	Mu-41	Turkey	Mucilage in the Sea of Marmara	Draft	4.52	61.6	90.0	116	-	99.9	0.00	
GCA_021440765.1	INSAq239	Portugal	Mussels	Draft	4.37	61.3	25.0	373	-	99.3	0.58	
GCA_026309555.1	CAPA008	Peru	*Oreochromis niloticus* kidney/liver, hemorrhagic disease	Draft	4.57	61.1	106.3	166	-	99.9	0.00	
GCA_026309515.1	CAPA022	Peru	*Oreochromis niloticus* kidney/liver, hemorrhagic disease	Draft	4.57	61.1	106.3	159	-	99.9	0.00	
GCA_024170045.1	SLBN-129	United States	*Oryza sativa*	Draft	4.47	61.9	4472.3	1	-	99.3	0.00	[20]
GCA_003849745.1	Aer593	Brazil	River	Draft	4.31	61.8	48.4	243	-	99.9	0.00	[21]
GCA_024649325.1	L215	China	River	Draft	4.50	61.5	73.9	152	-	99.9	0.00	
GCA_024649895.1	L7105	China	River	Draft	4.57	61.4	154.0	127	-	99.9	0.00	
GCA_024649655.1	L965	China	River	Draft	4.40	61.6	197.9	63	-	99.9	0.00	
GCA_024649525.1	M621	China	River	Draft	4.72	61.0	34.0	426	-	100.0	0.58	
GCA_024649925.1	S611	China	River	Draft	4.62	61.4	46.6	326	-	99.9	1.17	
GCA_900491645.1	ZJ17-2	China	River	Draft	4.56	61.3	59.7	257	-	100.0	0.00	[16]
GCA_900491695.1	ZJ66-1	China	River	Draft	4.39	61.5	75.8	153	-	100.0	0.00	[16]
GCA_001730215.1	CH129	Brazil	Seawater	Draft	4.42	61.6	156.4	63	-	99.9	0.00	[22]

GCA_007179295.1	WCW1-2	China	Sewage from a chicken farm	Complete	4.68	61.3	-	1	-	99.6	0.00	[23]
GCA_029961805.1	W3	China	Sludge	Complete	4.48	61.6		4	3	99.9	0.58	
GCA_004024475.1	GEO_23_Down_B	USA	Wastewater	Draft	4.51	61.6	36.6	270	-	99.7	0.00	[24]
GCA_009905865.1	GEO_37_Down_A	USA	Wastewater	Draft	4.46	61.4	69.2	167	-	99.7	0.00	[24]
GCA_004024195.1	GEO_47_Up_A	USA	Wastewater	Draft	4.56	61.6	34.9	243	-	99.9	0.58	[24]
GCA_004024325.1	GEO_48_Eff_A	USA	Wastewater	Draft	4.93	60.8	37.6	258	-	100.0	1.11	[24]
GCA_003925855.2	GSH8M-1	Japan	Wastewater	Complete	4.83	60.7	-	4	3	99.9	0.58	[25]
GCA_021609985.1	SCLZS52	China	Wastewater	Complete	4.94	61.3	-	9	8	100.0	0.58	[26]
GCA_937867955.1	SRR5626803_bin.50_CONCOCT_v1.1_MAG	South Africa	Wastewater	Draft	4.51	61.7	195.2	45	-	99.9	0.00	
GCA_013282845.1	TW-2	Poland	Wastewater	Draft	4.60	61.4	84.2	108	-	99.9	0.00	[27]
GCA_014168635.1	WP2-W18-ESBL-01	Japan	Wastewater	Complete	4.98	61.0	-	2	1	99.9	0.58	
GCA_014158455.1	WP3-S18-ESBL-02	Japan	Wastewater	Complete	4.86	61.6	-	1	-	99.9	0.58	
GCA_014169235.1	WP5-W18-ESBL-02	Japan	Wastewater	Complete	4.90	61.2	-	2	1	99.9	0.00	
GCA_014162015.1	WP8-S17-ESBL-03	Japan	Wastewater	Complete	4.35	61.7	-	1	-	99.9	0.00	
GCA_014169675.1	WP8-S18-CRE-01	Japan	Wastewater	Complete	4.83	60.7	-	4	3	99.9	0.58	
GCA_014169735.1	WP8-S18-ESBL-04	Japan	Wastewater	Complete	4.57	61.6	-	2	1	99.9	0.00	
GCA_003294925.2	R25-2	China	Wastewater sludge	Complete	5.01	60.7	-	3	2	99.2	0.44	[28]
GCA_003294855.2	R25-6	China	Wastewater sludge	Complete	4.71	61.4	-	2	1	99.9	0.00	[28]
GCA_003294895.2	T25-39	China	Wastewater sludge	Complete	4.97	60.7	-	3	2	100.0	0.44	[28]
GCA_006243135.1	TCO22	USA	Zebrafish gut	Draft	4.57	61.2	57.4	200	-	99.9	0.00	[29]
GCA_001730205.1	CHZ306	Brazil	Zooplankton from seawater	Draft	4.79	60.8	103.0	136	-	100.0	0.00	[30]
SRR14289142	AFW1-3	USA	Dairyfarm	Draft	4.40	61.8	120.4	69	-	99.9	0.00	[31]
SRR14289140	AFW5-2	USA	Dairyfarm	Draft	4.40	61.8	83.9	112	-	99.9	0.58	[31]
SRR14289133	AXW6-3w	USA	Dairyfarm	Draft	4.37	61.7	125.0	70	-	99.9	0.00	[31]
SRR14289139	AXS1-1	USA	Dairyfarm	Draft	4.40	61.8	114.9	76	-	99.9	0.00	[31]
SRR14289138	AXS1-4	USA	Dairyfarm	Draft	4.39	61.7	147.9	63	-	99.9	0.00	[31]
SRR14289135	AXS1-8-1	USA	Dairyfarm	Draft	4.36	61.8	138.8	65	-	99.9	0.00	[31]
SRR14289134	AXS1-8-2	USA	Dairyfarm	Draft	4.36	61.8	102.0	74	-	99.9	0.00	[31]
SRR14289137	AFT-1	USA	Dairyfarm	Draft	4.46	61.5	64.7	152	-	99.9	0.58	[31]
SRR14289136	AFT5	USA	Dairyfarm	Draft	4.40	61.8	110.4	72	-	99.9	0.00	[31]
SRR14289145	AFT6	USA	Dairyfarm	Draft	4.40	61.8	125.1	66	-	99.9	0.00	[31]

SRR14289144	AFT9	USA	Dairyfarm	Draft	4.51	61.4	98.0	83	-	99.9	0.00	[31]
SRR14289143	AFT9-5	USA	Dairyfarm	Draft	4.51	61.3	115.3	86	-	100.0	0.00	[31]
SRR14289149	AXT6	USA	Dairyfarm	Draft	4.40	61.8	120.3	65	-	99.9	0.00	[31]
SRR14289147	AXT9	USA	Dairyfarm	Draft	4.30	62.1	133.1	54	-	99.6	0.00	[31]
ERR3988471	UGB3607	France	Danio rerio larva	Draft	4.59	61.4	396.5	32	-	99.9	0.00	
DRR376747	STN211208-OZ-0m-04	Japan	Hospital sewage	Draft	4.99	60.5	32.5	413	-	100.0	0.00	
DRR376763	STN211208-OZ-10m-02	Japan	Hospital sewage	Draft	4.96	60.5	32.3	430	-	100.0	0.00	
DRR376768	STN211208-OZ-10m-07	Japan	Hospital sewage	Draft	4.81	60.8	27.1	379	-	100.0	0.00	
SRR22515265	AC95	China	Human ascites, chronic renal failure, peritonitis, diabetes, hypertension, renal anemia	Draft	4.35	61.8	248.3	38	-	99.9	0.00	
SRR22515306	AC108	China	Human bile, bile duct stones, cholangitis	Draft	4.31	61.7	87.5	123	-	99.9	0.29	
SRR22515297	AC148	China	Human bile, biliary mucinous cystadenoma	Draft	4.71	61.4	193.0	52	-	99.9	0.58	
SRR22515271	AC224	China	Human bile, cholangiocarcinoma	Draft	4.98	59.9	60.8	194	-	100.0	1.25	
SRR22515281	AC191	China	Human bile, cholangiocarcinoma, obstructive jaundice	Draft	4.36	62.0	102.1	91	-	99.9	0.00	
SRR22515303	AC117	China	Human bile, cholangiocarcinoma, obstructive jaundice	Draft	4.54	61.2	98.5	133	-	100.0	0.00	
SRR22515270	AC225	China	Human bile, cholangio-intestinal anastomotic stricture complicated by stones	Draft	4.49	61.5	248.2	56	-	99.9	0.00	
SRR22515277	AC74	China	Human bile, cholangitis, bile duct stones	Draft	4.27	61.9	153.9	78	-	99.9	0.29	

SRR22515268	AC92	China	Human bile, cholangitis, bile duct stones	Draft	4.27	62.0	353.3	34	-	99.9	0.00
SRR22515266	AC94	China	Human bile, choledochal cyst complicated by bile duct stones	Draft	4.37	61.9	258.5	46	-	99.9	0.00
SRR22515304	AC110	China	Human bile, choledochal cyst, biliary anastomotic obstruction	Draft	4.23	61.9	152.1	60	-	99.9	0.00
SRR22515309	AC98	China	Human bile, cystic dilatation of bile duct	Draft	4.37	61.5	67.3	144	-	99.9	0.29
SRR22515302	AC122	China	Human bile, liver cancer, obstructive jaundice	Draft	4.50	61.5	204.5	66	-	99.6	0.00
SRR22515285	AC181	China	Human bile, pancreatic head carcinoma	Draft	4.35	61.8	125.7	74	-	99.9	0.00
SRR22515284	AC184	China	Human bile, rectal cancer	Draft	4.49	61.3	66.3	177	-	100.0	0.00
SRR22515301	AC128	China	Human blood	Draft	4.50	61.5	176.6	72	-	99.6	0.00
SRR24749619	2021XX-00002	USA	Human blood	Draft	4.63	61.3	36.0	225	-	99.9	0.44
SRR22515279	AC193	China	Human blood, bile duct stones	Draft	4.38	62.0	203.2	46	-	99.9	0.00
SRR22515267	AC93	China	Human blood, bile duct stones	Draft	4.38	61.5	111.4	98	-	99.9	0.00
SRR22515272	AC222	China	Human blood, cholangiocarcinoma, abdominal infection	Draft	4.37	61.7	373.0	38	-	99.9	0.00
SRR22515273	AC207	China	Human blood, cholangiocarcinoma, cholangio-intestinal anastomotic stricture, cholangitis	Draft	4.47	61.5	264.4	39	-	99.9	0.00
SRR22515276	AC197	China	Human blood, cholangitis, bile duct stones	Draft	4.45	61.7	54.5	194	-	99.9	0.00

SRR22515311	AC69	China	Human blood, cholangitis, pancreatic cancer, biliary tract obstruction	Draft	4.33	62.0	363.5	32	-	99.9	0.00
SRR22515296	AC151	China	Human blood, chronic obstructive pulmonary disease	Draft	4.42	61.5	80.3	153	-	99.9	0.00
SRR22515291	AC165	China	Human blood, gallbladder stones	Draft	4.40	62.0	374.6	36	-	99.9	0.00
SRR22515290	AC168	China	Human blood, gallbladder stones	Draft	4.43	61.5	88.0	94	-	99.9	0.00
SRR22515293	AC158	China	Human blood, gallbladder stones, coronary atherosclerotic heart disease	Draft	4.59	61.2	167.8	72	-	99.9	0.00
SRR22515308	AC100	China	Human blood, lung cancer	Draft	4.34	62.0	220.4	34	-	99.9	0.00
SRR22515307	AC106	China	Human blood, lung cancer, hypertension, diabetes	Draft	4.55	61.8	170.4	44	-	99.9	0.00
SRR11247323	600558	Bangladesh	Human faeces, diarrhea	Draft	4.24	61.8	139.0	68	-	99.3	0.15
SRR11247259	600105	Bangladesh	Human faeces, diarrhea	Draft	4.46	61.3	53.0	168	-	100.0	0.00
SRR11247484	600142	Bangladesh	Human faeces, diarrhea	Draft	4.37	61.8	243.0	47	-	99.9	0.00
SRR11247442	600199	Bangladesh	Human faeces, diarrhea	Draft	4.38	61.9	184.6	63	-	99.9	0.00
SRR11247215	600277	Bangladesh	Human faeces, diarrhea	Draft	4.25	62.0	189.8	48	-	99.9	0.00
SRR11247181	600295	Bangladesh	Human faeces, diarrhea	Draft	4.22	61.9	104.2	97	-	99.9	0.00
SRR11247157	600334	Bangladesh	Human faeces, diarrhea	Draft	4.38	61.8	271.8	40	-	99.9	0.00
SRR11247124	600358	Bangladesh	Human faeces, diarrhea	Draft	4.47	61.8	185.2	48	-	99.6	0.00

SRR11247102	600406	Bangladesh	Human faeces, diarrhea	Draft	4.27	61.7	98.2	82	-	99.9	0.00
SRR11247090	600430	Bangladesh	Human faeces, diarrhea	Draft	4.36	62.1	188.5	45	-	99.9	0.00
SRR11247368	600518	Bangladesh	Human faeces, diarrhea	Draft	4.44	61.4	71.9	146	-	99.9	0.00
SRR11247357	600521	Bangladesh	Human faeces, diarrhea	Draft	4.30	61.9	71.8	117	-	99.9	0.00
SRR11247285	600590	Bangladesh	Human faeces, diarrhea	Draft	4.33	61.5	106.7	104	-	99.9	0.00
SRR11247277	600744	Bangladesh	Human faeces, diarrhea	Draft	4.32	61.7	151.2	75	-	99.3	0.00
SRR11247271	600812	Bangladesh	Human faeces, diarrhea	Draft	4.34	61.9	324.8	28	-	99.9	0.00
SRR11247268	600856	Bangladesh	Human faeces, diarrhea	Draft	4.31	61.8	172.4	64	-	99.9	0.00
SRR11247267	600866	Bangladesh	Human faeces, diarrhea	Draft	4.36	61.8	154.3	69	-	99.9	0.00
SRR11247265	600910	Bangladesh	Human faeces, diarrhea	Draft	4.33	61.9	134.6	76	-	99.9	0.00
SRR11247264	600932	Bangladesh	Human faeces, diarrhea	Draft	4.26	61.8	136.3	62	-	99.9	0.00
SRR11247262	600960	Bangladesh	Human faeces, diarrhea	Draft	4.45	62.1	344.3	33	-	99.9	0.00
SRR11247260	600965	Bangladesh	Human faeces, diarrhea	Draft	4.31	61.6	141.8	88	-	99.3	0.00
SRR11247257	600973	Bangladesh	Human faeces, diarrhea	Draft	4.40	61.8	358.9	29	-	99.3	0.00
SRR11247254	600989	Bangladesh	Human faeces, diarrhea	Draft	4.34	61.8	371.9	32	-	99.9	0.00
SRR11247252	601023	Bangladesh	Human faeces, diarrhea	Draft	4.31	61.8	227.9	46	-	99.9	0.00
SRR11247505	601060	Bangladesh	Human faeces, diarrhea	Draft	4.34	61.8	180.3	57	-	99.9	0.00
SRR11247502	601085	Bangladesh	Human faeces, diarrhea	Draft	4.34	62.0	313.3	42	-	99.3	0.00
SRR11247494	601149	Bangladesh	Human faeces, diarrhea	Draft	4.30	61.9	252.7	41	-	99.6	0.00

SRR11247483	601294	Bangladesh	Human faeces, diarrhea	Draft	4.35	61.9	65.2	144	-	99.9	0.00
SRR11247481	602018	Bangladesh	Human faeces, diarrhea	Draft	4.35	61.8	273.6	33	-	99.9	0.00
SRR11247479	602193	Bangladesh	Human faeces, diarrhea	Draft	4.39	61.4	55.1	169	-	100.0	0.00
SRR11247471	602312	Bangladesh	Human faeces, diarrhea	Draft	4.37	61.6	204.5	60	-	99.9	0.00
SRR11247468	602416	Bangladesh	Human faeces, diarrhea	Draft	4.39	61.4	90.9	141	-	99.9	0.00
SRR11247467	602429	Bangladesh	Human faeces, diarrhea	Draft	4.35	61.5	112.4	79	-	99.9	0.29
SRR11247466	602432	Bangladesh	Human faeces, diarrhea	Draft	4.43	61.7	306.9	36	-	99.6	0.00
SRR11247465	602434	Bangladesh	Human faeces, diarrhea	Draft	4.29	61.7	134.5	86	-	99.9	0.00
SRR11247464	602438	Bangladesh	Human faeces, diarrhea	Draft	4.29	61.9	131.8	75	-	99.3	0.00
SRR11247463	602443	Bangladesh	Human faeces, diarrhea	Draft	4.29	61.8	226.1	45	-	99.9	0.00
SRR11247170	602491	Bangladesh	Human faeces, diarrhea	Draft	4.34	62.0	130.9	58	-	99.9	0.00
SRR11247169	602497	Bangladesh	Human faeces, diarrhea	Draft	4.29	62.1	188.0	48	-	99.9	0.00
SRR11247458	602511	Bangladesh	Human faeces, diarrhea	Draft	4.20	62.0	55.5	173	-	99.9	0.00
SRR11247457	602512	Bangladesh	Human faeces, diarrhea	Draft	4.88	60.5	88.9	140	-	100.0	0.78
SRR11247456	602521	Bangladesh	Human faeces, diarrhea	Draft	4.23	61.8	91.4	99	-	99.9	0.00
SRR11247451	602596	Bangladesh	Human faeces, diarrhea	Draft	4.41	61.5	98.5	99	-	100.0	0.00
SRR11247449	602607	Bangladesh	Human faeces, diarrhea	Draft	4.39	61.4	49.1	206	-	99.9	0.00
SRR11247448	602614	Bangladesh	Human faeces, diarrhea	Draft	4.31	61.8	181.6	54	-	99.3	0.00
SRR11247447	602633	Bangladesh	Human faeces, diarrhea	Draft	4.40	61.6	86.0	116	-	99.9	0.00

SRR11247444	602682	Bangladesh	Human faeces, diarrhea	Draft	4.68	61.2	139.2	105	-	100.0	0.44
SRR11247443	602686	Bangladesh	Human faeces, diarrhea	Draft	4.40	61.7	173.2	74	-	99.9	0.00
SRR11247441	602710	Bangladesh	Human faeces, diarrhea	Draft	4.41	61.6	63.2	159	-	99.9	0.00
SRR11247440	602747	Bangladesh	Human faeces, diarrhea	Draft	4.41	61.7	101.7	112	-	99.3	0.58
SRR11247439	602757	Bangladesh	Human faeces, diarrhea	Draft	4.38	62.0	304.2	31	-	99.9	0.00
SRR11247437	602762	Bangladesh	Human faeces, diarrhea	Draft	4.36	61.3	59.3	167	-	99.9	0.00
SRR11247436	602763	Bangladesh	Human faeces, diarrhea	Draft	4.37	61.6	92.7	108	-	99.9	0.00
SRR11247435	602764	Bangladesh	Human faeces, diarrhea	Draft	4.25	61.8	121.7	70	-	99.9	0.00
SRR11247434	602765	Bangladesh	Human faeces, diarrhea	Draft	4.37	61.7	88.6	107	-	99.9	0.00
SRR11247432	602768	Bangladesh	Human faeces, diarrhea	Draft	4.34	61.8	162.0	49	-	99.9	0.58
SRR11247430	602784	Bangladesh	Human faeces, diarrhea	Draft	4.36	62.0	179.0	55	-	99.9	0.00
SRR11247429	602809	Bangladesh	Human faeces, diarrhea	Draft	4.30	61.7	248.2	56	-	99.9	0.29
SRR11247427	602854	Bangladesh	Human faeces, diarrhea	Draft	4.36	61.4	70.7	153	-	100.0	0.00
SRR11247425	602900	Bangladesh	Human faeces, diarrhea	Draft	4.60	60.9	74.2	167	-	100.0	0.00
SRR11247424	602905	Bangladesh	Human faeces, diarrhea	Draft	4.34	61.9	111.9	84	-	99.9	0.00
SRR11247249	602931	Bangladesh	Human faeces, diarrhea	Draft	4.76	60.5	73.0	161	-	100.0	0.78
SRR11247246	602962	Bangladesh	Human faeces, diarrhea	Draft	4.26	61.9	120.5	73	-	99.9	0.00
SRR11247245	602985	Bangladesh	Human faeces, diarrhea	Draft	4.34	62.0	296.8	33	-	99.9	0.00
SRR11247243	603002	Bangladesh	Human faeces, diarrhea	Draft	4.49	61.3	77.7	160	-	99.9	0.00

SRR11247241	603039	Bangladesh	Human faeces, diarrhea	Draft	4.39	61.9	326.9	29	-	99.9	0.00
SRR11247240	603040	Bangladesh	Human faeces, diarrhea	Draft	4.53	61.7	413.8	44	-	99.9	0.00
SRR11247239	603041	Bangladesh	Human faeces, diarrhea	Draft	4.36	61.5	102.0	99	-	100.0	0.00
SRR11247238	603047	Bangladesh	Human faeces, diarrhea	Draft	4.87	60.2	62.2	168	-	100.0	0.67
SRR11247232	603114	Bangladesh	Human faeces, diarrhea	Draft	4.43	61.3	55.9	169	-	100.0	0.00
SRR11247228	603246	Bangladesh	Human faeces, diarrhea	Draft	4.37	61.7	144.3	65	-	99.9	0.00
SRR11247227	603253	Bangladesh	Human faeces, diarrhea	Draft	4.36	61.6	90.6	143	-	99.9	0.00
SRR11247224	603287	Bangladesh	Human faeces, diarrhea	Draft	4.45	61.5	93.1	101	-	100.0	0.00
SRR11247222	603316	Bangladesh	Human faeces, diarrhea	Draft	4.31	62.1	320.0	37	-	99.9	0.00
SRR11247220	603360	Bangladesh	Human faeces, diarrhea	Draft	4.23	62.0	171.2	65	-	99.9	0.00
SRR11247217	603392	Bangladesh	Human faeces, diarrhea	Draft	4.31	61.8	53.3	177	-	99.9	0.00
SRR11247213	603465	Bangladesh	Human faeces, diarrhea	Draft	4.34	62.0	400.6	19	-	99.9	0.00
SRR11247212	603539	Bangladesh	Human faeces, diarrhea	Draft	4.44	61.4	62.7	163	-	99.9	0.00
SRR11247209	603573	Bangladesh	Human faeces, diarrhea	Draft	4.28	62.0	379.0	32	-	99.9	0.00
SRR11247208	603579	Bangladesh	Human faeces, diarrhea	Draft	4.43	61.6	107.5	106	-	99.9	0.00
SRR11247207	603595	Bangladesh	Human faeces, diarrhea	Draft	4.41	61.5	103.3	96	-	99.9	0.00
SRR11247200	603625	Bangladesh	Human faeces, diarrhea	Draft	4.28	61.8	95.3	79	-	99.9	0.29
SRR11247198	603650	Bangladesh	Human faeces, diarrhea	Draft	4.34	61.6	46.4	194	-	99.9	0.00
SRR11247194	603688	Bangladesh	Human faeces, diarrhea	Draft	4.35	61.8	212.5	55	-	99.9	0.00

SRR11247190	603709	Bangladesh	Human faeces, diarrhea	Draft	4.37	62.0	114.0	88	-	99.9	0.00
SRR11247188	603746	Bangladesh	Human faeces, diarrhea	Draft	4.27	62.0	151.6	56	-	99.9	0.00
SRR11247183	603761	Bangladesh	Human faeces, diarrhea	Draft	4.40	62.0	322.8	26	-	99.9	0.00
SRR11247182	603822	Bangladesh	Human faeces, diarrhea	Draft	4.34	61.6	76.8	160	-	99.9	0.00
SRR11247180	603869	Bangladesh	Human faeces, diarrhea	Draft	4.41	61.8	344.6	28	-	99.9	0.00
SRR11247179	603899	Bangladesh	Human faeces, diarrhea	Draft	4.39	62.0	261.6	32	-	99.9	0.00
SRR11247178	603901	Bangladesh	Human faeces, diarrhea	Draft	4.40	61.3	57.5	165	-	99.9	0.00
SRR11247177	603917	Bangladesh	Human faeces, diarrhea	Draft	4.35	61.8	185.4	48	-	99.9	0.00
SRR11247175	603962	Bangladesh	Human faeces, diarrhea	Draft	4.33	61.8	186.6	53	-	99.9	0.00
SRR11247173	604001	Bangladesh	Human faeces, diarrhea	Draft	4.31	61.6	177.7	52	-	99.9	0.00
SRR11247172	604002	Bangladesh	Human faeces, diarrhea	Draft	4.26	61.9	173.5	57	-	99.9	0.00
SRR11247167	604018	Bangladesh	Human faeces, diarrhea	Draft	4.45	61.5	70.5	128	-	99.9	0.00
SRR11247166	604022	Bangladesh	Human faeces, diarrhea	Draft	4.31	61.9	141.3	79	-	99.3	0.00
SRR11247165	604053	Bangladesh	Human faeces, diarrhea	Draft	4.47	61.4	70.7	177	-	99.9	0.00
SRR11247164	604054	Bangladesh	Human faeces, diarrhea	Draft	4.30	61.6	123.4	75	-	99.9	0.00
SRR11247163	604074	Bangladesh	Human faeces, diarrhea	Draft	4.36	62.0	199.9	42	-	99.9	0.00
SRR11247162	604081	Bangladesh	Human faeces, diarrhea	Draft	4.46	61.5	128.8	86	-	99.9	0.00
SRR11247160	604096	Bangladesh	Human faeces, diarrhea	Draft	4.34	61.6	100.3	88	-	100.0	0.00
SRR11247159	604124	Bangladesh	Human faeces, diarrhea	Draft	4.58	61.3	86.5	93	-	100.0	0.58

SRR11247158	604136	Bangladesh	Human faeces, diarrhea	Draft	4.51	61.4	151.1	102	-	99.9	0.00
SRR11247156	604141	Bangladesh	Human faeces, diarrhea	Draft	4.48	61.3	48.1	221	-	99.9	0.00
SRR11247155	604142	Bangladesh	Human faeces, diarrhea	Draft	4.41	61.4	62.0	151	-	99.9	0.00
SRR11247153	604185	Bangladesh	Human faeces, diarrhea	Draft	4.33	61.8	147.3	52	-	99.9	0.00
SRR11247152	604193	Bangladesh	Human faeces, diarrhea	Draft	4.47	61.4	81.5	152	-	99.9	0.00
SRR11247149	604270	Bangladesh	Human faeces, diarrhea	Draft	4.45	61.9	369.7	35	-	99.9	0.00
SRR11247145	604281	Bangladesh	Human faeces, diarrhea	Draft	4.54	61.3	59.5	161	-	99.9	0.00
SRR11247143	604295	Bangladesh	Human faeces, diarrhea	Draft	4.30	61.9	160.2	63	-	99.9	0.00
SRR11247142	604329	Bangladesh	Human faeces, diarrhea	Draft	4.43	61.6	203.1	50	-	99.9	0.00
SRR11247141	604333	Bangladesh	Human faeces, diarrhea	Draft	4.41	61.4	122.7	81	-	99.9	0.29
SRR11247140	604339	Bangladesh	Human faeces, diarrhea	Draft	4.37	61.5	101.0	79	-	99.9	0.00
SRR11247138	604370	Bangladesh	Human faeces, diarrhea	Draft	4.25	62.0	214.7	55	-	99.9	0.00
SRR11247137	604381	Bangladesh	Human faeces, diarrhea	Draft	4.49	61.6	220.7	38	-	99.9	0.00
SRR11247127	604469	Bangladesh	Human faeces, diarrhea	Draft	4.46	61.4	70.3	140	-	99.9	0.29
SRR11247122	604500	Bangladesh	Human faeces, diarrhea	Draft	4.50	61.5	89.4	122	-	99.9	0.00
SRR11247121	604504	Bangladesh	Human faeces, diarrhea	Draft	4.30	62.2	396.7	28	-	99.9	0.00
SRR11247120	604518	Bangladesh	Human faeces, diarrhea	Draft	4.33	61.7	127.1	97	-	99.9	0.00
SRR11247119	604533	Bangladesh	Human faeces, diarrhea	Draft	4.37	61.8	234.5	37	-	99.9	0.00
SRR11247117	604542	Bangladesh	Human faeces, diarrhea	Draft	4.45	61.4	50.4	183	-	100.0	0.00

SRR11247313	A600056	Bangladesh	Human faeces, diarrhea	Draft	4.35	62.1	396.1	28	-	99.9	0.00
SRR11247310	A600239	Bangladesh	Human faeces, diarrhea	Draft	4.52	61.4	95.6	104	-	99.9	0.00
SRR11247309	A600241	Bangladesh	Human faeces, diarrhea	Draft	4.42	61.5	106.7	90	-	99.9	0.00
SRR11247307	A600480	Bangladesh	Human faeces, diarrhea	Draft	4.33	62.0	173.4	51	-	99.9	0.00
SRR11247305	A600566	Bangladesh	Human faeces, diarrhea	Draft	4.31	61.8	199.9	49	-	99.9	0.00
SRR11247303	A600673	Bangladesh	Human faeces, diarrhea	Draft	4.44	62.0	200.9	41	-	99.9	0.58
SRR11247302	A600716	Bangladesh	Human faeces, diarrhea	Draft	4.31	62.0	184.4	56	-	99.9	0.00
SRR11247299	A600749	Bangladesh	Human faeces, diarrhea	Draft	4.31	61.6	104.2	80	-	99.9	0.00
SRR11247297	A600919	Bangladesh	Human faeces, diarrhea	Draft	4.27	61.9	217.5	47	-	99.9	0.00
SRR11247231	A603171	Bangladesh	Human faeces, diarrhea	Draft	4.31	61.8	241.7	57	-	99.9	0.00
SRR11247486	500116	India	Human faeces, diarrhea	Draft	4.72	60.3	107.6	92	-	100.0	0.67
SRR11247485	500297	India	Human faeces, diarrhea	Draft	4.41	61.4	134.7	72	-	99.9	0.00
SRR11247091	503237	India	Human faeces, diarrhea	Draft	4.47	61.8	104.9	118	-	99.9	0.58
SRR11247281	503742	India	Human faeces, diarrhea	Draft	4.40	61.4	146.6	78	-	99.9	0.00
SRR11247082	700555	Pakistan	Human faeces, diarrhea	Draft	4.43	61.4	167.7	47	-	99.6	0.00
SRR11247115	700006	Pakistan	Human faeces, diarrhea	Draft	4.43	61.3	52.9	203	-	99.9	0.00
SRR11247111	700049	Pakistan	Human faeces, diarrhea	Draft	4.47	61.4	129.4	92	-	99.9	0.00
SRR11247109	700053	Pakistan	Human faeces, diarrhea	Draft	4.48	61.4	55.9	147	-	100.0	0.00
SRR11247107	700078	Pakistan	Human faeces, diarrhea	Draft	4.31	61.7	51.3	170	-	99.9	0.19

SRR11247106	700092	Pakistan	Human faeces, diarrhea	Draft	4.39	61.5	80.7	100	-	99.9	0.00
SRR11247105	700144	Pakistan	Human faeces, diarrhea	Draft	4.26	62.0	113.6	84	-	99.9	0.00
SRR11247104	700172	Pakistan	Human faeces, diarrhea	Draft	4.38	62.0	401.4	28	-	99.9	0.00
SRR11247099	700302	Pakistan	Human faeces, diarrhea	Draft	4.39	61.4	53.6	188	-	99.3	0.00
SRR11247096	700335	Pakistan	Human faeces, diarrhea	Draft	4.38	61.6	96.1	105	-	99.9	0.00
SRR11247095	700339	Pakistan	Human faeces, diarrhea	Draft	4.31	62.0	173.1	46	-	99.3	0.00
SRR11247094	700355	Pakistan	Human faeces, diarrhea	Draft	4.23	61.9	96.5	95	-	99.9	0.00
SRR11247092	700380	Pakistan	Human faeces, diarrhea	Draft	4.32	61.8	77.3	116	-	99.9	0.00
SRR11247088	700394	Pakistan	Human faeces, diarrhea	Draft	4.36	61.4	53.7	178	-	99.9	0.29
SRR11247087	700481	Pakistan	Human faeces, diarrhea	Draft	4.37	61.6	48.2	185	-	99.9	0.00
SRR11247085	700493	Pakistan	Human faeces, diarrhea	Draft	4.31	61.8	123.5	83	-	99.9	0.00
SRR11247080	700684	Pakistan	Human faeces, diarrhea	Draft	4.37	61.5	118.1	85	-	99.9	0.00
SRR11247420	700701	Pakistan	Human faeces, diarrhea	Draft	4.40	61.3	96.8	109	-	99.9	0.29
SRR11247418	700762	Pakistan	Human faeces, diarrhea	Draft	4.41	61.3	89.6	135	-	99.9	0.29
SRR11247417	700783	Pakistan	Human faeces, diarrhea	Draft	4.33	61.9	102.4	79	-	99.9	0.00
SRR11247414	700818	Pakistan	Human faeces, diarrhea	Draft	4.43	61.3	70.3	138	-	99.9	0.00
SRR11247413	700991	Pakistan	Human faeces, diarrhea	Draft	4.22	61.9	278.6	38	-	99.9	0.00
SRR11247411	702008	Pakistan	Human faeces, diarrhea	Draft	4.41	61.9	303.8	34	-	99.9	0.00
SRR11247410	702050	Pakistan	Human faeces, diarrhea	Draft	4.43	62.0	341.1	42	-	99.9	0.00

SRR11247409	702051	Pakistan	Human faeces, diarrhea	Draft	4.44	61.5	87.7	123	-	99.9	0.00
SRR11247406	702154	Pakistan	Human faeces, diarrhea	Draft	4.43	61.3	60.9	152	-	99.9	0.00
SRR11247405	702186	Pakistan	Human faeces, diarrhea	Draft	4.49	61.5	57.5	201	-	99.9	0.00
SRR11247404	702210	Pakistan	Human faeces, diarrhea	Draft	4.40	61.9	328.8	36	-	99.9	0.00
SRR11247403	702277	Pakistan	Human faeces, diarrhea	Draft	4.41	61.4	90.6	129	-	99.9	0.00
SRR11247399	702332	Pakistan	Human faeces, diarrhea	Draft	4.46	61.8	65.4	135	-	99.9	0.00
SRR11247398	702427	Pakistan	Human faeces, diarrhea	Draft	4.39	61.9	218.6	47	-	99.9	0.00
SRR11247397	702428	Pakistan	Human faeces, diarrhea	Draft	4.40	61.9	100.8	94	-	99.9	0.00
SRR11247396	702434	Pakistan	Human faeces, diarrhea	Draft	4.39	61.9	54.2	146	-	99.9	0.00
SRR11247395	702464	Pakistan	Human faeces, diarrhea	Draft	4.40	61.9	192.7	47	-	99.9	0.00
SRR11247394	702465	Pakistan	Human faeces, diarrhea	Draft	4.41	61.9	416.8	34	-	99.9	0.00
SRR11247393	702469	Pakistan	Human faeces, diarrhea	Draft	4.41	61.9	299.1	35	-	99.9	0.00
SRR11247392	702496	Pakistan	Human faeces, diarrhea	Draft	4.41	61.9	229.6	37	-	99.9	0.00
SRR11247391	702540	Pakistan	Human faeces, diarrhea	Draft	4.54	61.1	61.4	182	-	99.9	0.00
SRR11247389	702552	Pakistan	Human faeces, diarrhea	Draft	4.36	61.9	52.9	197	-	99.9	0.00
SRR11247388	702590	Pakistan	Human faeces, diarrhea	Draft	4.41	61.9	292.4	38	-	99.9	0.00
SRR11247387	702606	Pakistan	Human faeces, diarrhea	Draft	4.40	61.9	186.1	55	-	99.9	0.00
SRR11247386	702615	Pakistan	Human faeces, diarrhea	Draft	4.37	62.0	70.9	125	-	99.9	0.19
SRR11247385	702658	Pakistan	Human faeces, diarrhea	Draft	4.42	61.5	68.7	155	-	99.9	0.00

SRR11247384	702699	Pakistan	Human faeces, diarrhea	Draft	4.46	61.9	183.7	51	-	99.9	0.00
SRR11247383	702711	Pakistan	Human faeces, diarrhea	Draft	4.32	61.9	205.6	46	-	99.9	0.00
SRR11247380	702831	Pakistan	Human faeces, diarrhea	Draft	4.37	61.7	61.2	165	-	99.9	0.00
SRR11247376	702871	Pakistan	Human faeces, diarrhea	Draft	4.25	61.8	57.8	151	-	99.9	0.00
SRR11247375	702903	Pakistan	Human faeces, diarrhea	Draft	4.38	61.9	149.9	55	-	99.9	0.00
SRR11247374	702925	Pakistan	Human faeces, diarrhea	Draft	4.33	62.1	218.8	39	-	99.6	0.00
SRR11247372	703040	Pakistan	Human faeces, diarrhea	Draft	4.40	61.6	73.4	168	-	99.9	0.00
SRR11247371	703046	Pakistan	Human faeces, diarrhea	Draft	4.55	61.2	75.0	127	-	99.9	0.00
SRR11247370	703054	Pakistan	Human faeces, diarrhea	Draft	4.49	61.3	25.0	346	-	99.9	0.00
SRR11247369	703076	Pakistan	Human faeces, diarrhea	Draft	4.58	61.2	82.7	129	-	99.9	0.58
SRR11247367	703089	Pakistan	Human faeces, diarrhea	Draft	4.35	61.6	215.3	65	-	99.3	0.00
SRR11247366	703097	Pakistan	Human faeces, diarrhea	Draft	4.28	61.8	140.5	64	-	99.9	0.00
SRR11247365	703129	Pakistan	Human faeces, diarrhea	Draft	4.43	61.5	90.3	137	-	99.9	1.17
SRR11247364	703142	Pakistan	Human faeces, diarrhea	Draft	4.37	62.0	50.4	200	-	99.9	0.00
SRR11247363	703164	Pakistan	Human faeces, diarrhea	Draft	4.50	61.2	79.3	165	-	99.9	0.00
SRR11247360	703236	Pakistan	Human faeces, diarrhea	Draft	4.42	61.5	76.4	131	-	99.9	0.00
SRR11247358	703254	Pakistan	Human faeces, diarrhea	Draft	4.46	61.4	72.7	133	-	99.9	0.00
SRR11247353	703317	Pakistan	Human faeces, diarrhea	Draft	4.38	62.0	126.1	86	-	99.9	0.00
SRR11247351	703340	Pakistan	Human faeces, diarrhea	Draft	4.34	62.1	152.7	54	-	99.9	0.00

SRR11247349	703372	Pakistan	Human faeces, diarrhea	Draft	4.40	61.7	115.8	87	-	99.9	0.29
SRR11247347	703439	Pakistan	Human faeces, diarrhea	Draft	4.48	61.7	264.3	38	-	99.9	0.58
SRR11247345	703472	Pakistan	Human faeces, diarrhea	Draft	4.44	61.3	50.0	186	-	99.9	0.00
SRR11247343	703524	Pakistan	Human faeces, diarrhea	Draft	4.43	61.3	67.0	157	-	99.9	0.29
SRR11247342	703530	Pakistan	Human faeces, diarrhea	Draft	4.37	61.8	100.9	119	-	99.9	0.00
SRR11247341	703548	Pakistan	Human faeces, diarrhea	Draft	4.36	62.0	407.2	30	-	99.9	0.00
SRR11247339	703603	Pakistan	Human faeces, diarrhea	Draft	4.45	61.6	185.1	54	-	99.9	0.00
SRR11247338	703620	Pakistan	Human faeces, diarrhea	Draft	4.42	61.3	92.4	100	-	99.9	0.29
SRR11247336	703636	Pakistan	Human faeces, diarrhea	Draft	4.41	61.9	57.2	166	-	99.9	0.00
SRR11247334	703667	Pakistan	Human faeces, diarrhea	Draft	4.41	61.4	51.6	241	-	99.9	0.00
SRR11247332	703819	Pakistan	Human faeces, diarrhea	Draft	4.25	61.7	32.3	304	-	99.3	0.00
SRR11247331	703853	Pakistan	Human faeces, diarrhea	Draft	4.28	61.8	105.6	93	-	99.9	0.00
SRR11247329	703914	Pakistan	Human faeces, diarrhea	Draft	4.48	61.6	204.5	51	-	99.9	0.00
SRR11247327	704013	Pakistan	Human faeces, diarrhea	Draft	4.42	61.5	48.6	165	-	99.9	0.00
SRR11247321	704081	Pakistan	Human faeces, diarrhea	Draft	4.54	61.2	96.6	130	-	99.9	0.00
SRR11247320	704099	Pakistan	Human faeces, diarrhea	Draft	4.30	61.8	148.4	66	-	99.9	0.00
SRR11247319	704116	Pakistan	Human faeces, diarrhea	Draft	4.51	61.3	207.1	51	-	99.9	0.00
SRR11247317	704158	Pakistan	Human faeces, diarrhea	Draft	4.28	61.9	167.7	47	-	99.9	0.00
SRR11247316	704205	Pakistan	Human faeces, diarrhea	Draft	4.60	61.2	76.1	163	-	99.9	0.00

SRR11247314	704263	Pakistan	Human faeces, diarrhea	Draft	4.47	61.3	58.2	171	-	99.9	0.00	
SRR11247116	A700004	Pakistan	Human faeces, diarrhea	Draft	4.35	61.5	62.5	152	-	99.9	0.00	
SRR11247100	A700241	Pakistan	Human faeces, diarrhea	Draft	4.38	62.0	300.5	33	-	99.9	0.00	
SRR11247097	A700329	Pakistan	Human faeces, diarrhea	Draft	4.36	61.7	43.8	245	-	99.9	0.00	
SRR11247292	A700868	Pakistan	Human faeces, diarrhea	Draft	4.41	61.5	105.3	102	-	100.0	0.00	
SRR11247291	A702252	Pakistan	Human faeces, diarrhea	Draft	4.28	61.9	96.0	100	-	99.9	0.88	
SRR11247402	A702278	Pakistan	Human faeces, diarrhea	Draft	4.49	61.5	51.9	213	-	99.9	0.00	
SRR11247400	A702287	Pakistan	Human faeces, diarrhea	Draft	4.46	61.3	124.3	88	-	99.9	0.00	
SRR11247362	A703210	Pakistan	Human faeces, diarrhea	Draft	4.33	61.6	131.2	70	-	99.9	0.00	
SRR11247355	A703275	Pakistan	Human faeces, diarrhea	Draft	4.35	61.6	177.7	66	-	99.3	0.00	
SRR11247288	A703724_2	Pakistan	Human faeces, diarrhea	Draft	4.34	61.8	107.7	79	-	99.9	0.00	
ERR10441321	DTU_2020_1001138_1_SI_CAN_EDM_051B	Canada	Human faeces, diseased	Draft	4.50	61.2	29.7	262	-	99.9	0.88	
SRR22515280	AC192	China	Human sputum coronary atherosclerotic heart disease, hypertension + *Pseudomonas aeruginosa*	Draft	4.58	61.2	77.4	184	-	99.9	0.00	[32]
SRR22515300	AC144	China	Human sputum esophageal cancer + *Pseudomonas aeruginosa*	Draft	4.62	61.1	61.0	214	-	99.9	0.58	[32]
SRR22515298	AC145	China	Human sputum gallbladder stones,	Draft	4.52	61.6	197.4	49	-	99.9	0.58	[32]

			cholecystitis, pancreatitis									
SRR22515295	AC154	China	Human sputum gastrointestinal hemorrhage, hypertension, renal insufficiency	Draft	4.72	61.4	103.5	99	-	99.9	0.00	[32]
SRR22515299	AC71	China	Human sputum hypertension, coronoary atherosclerotic heart disease	Draft	4.62	61.3	60.2	192	-	99.9	0.00	[32]
SRR22515283	AC186	China	Human sputum, cardiac insufficiency	Draft	4.76	61.2	68.4	188	-	100.0	0.00	[32]
SRR22515278	AC194	China	Human sputum, left lung shadow	Draft	4.44	61.4	105.0	132	-	99.9	0.88	[32]
SRR22515310	AC70	China	Human urine, connective tissue disease, cirrhosis, autoimmune hemolytic anemia	Draft	4.62	61.3	66.4	212	-	99.9	0.58	[32]
SRR22515288	AC73	China	Human urine, postoperative of ureteral stones	Draft	4.54	61.7	263.5	57	-	99.9	0.00	[32]
SRR22515289	AC169	China	Human urine, post-prostate paracentesis, urinary tract infection	Draft	4.88	61.0	72.3	170	-	100.0	0.44	[32]
SRR22515269	AC82	China	Human urine, urethral atresia, post-cystostomy	Draft	4.38	62.0	215.2	41	-	99.9	0.00	[32]
SRR22515282	AC189	China	Human urine, urinary retention, prostatic hyperplasia	Draft	4.48	61.6	137.3	85	-	99.9	0.00	[32]
SRR22515294	AC156	China	Human urine, vertebrobasilar artery stenosis	Draft	4.47	61.4	171.2	76	-	99.9	0.00	[32]
SRR19859888	A2_3	South Africa	River	Draft	4.61	61.3	28.6	286	-	99.9	0.00	[33]
SRR19859879	A4_15	South Africa	River	Draft	4.77	61.3	53.1	203	-	99.9	0.00	[33]

SRR19859883	A2_12	South Africa	River	Draft	4.95	61.1	33.8	367	-	100.0	1.02	[33]
SRR19859868	A5_12	South Africa	River	Draft	5.28	60.2	25.6	428	-	100.0	1.02	[33]
SRR19859867	A5_13	South Africa	River	Draft	4.70	61.0	24.5	417	-	100.0	1.17	[33]
SRR19859866	A5_14	South Africa	River	Draft	5.18	60.6	20.6	498	-	100.0	1.02	[33]
SRR19859855	A6_11	South Africa	River	Draft	5.13	60.5	21.4	457	-	100.0	1.02	[33]
SRR19859834	A7_3	South Africa	River	Draft	4.66	61.1	32.3	305	-	100.0	0.00	[33]
SRR19859802	B6_25	South Africa	River	Draft	4.54	61.4	65.2	143	-	99.9	0.00	[33]
SRR19859908	S4_3	South Africa	River	Draft	4.71	61.1	23.3	374	-	99.9	1.17	[33]
SRR19859798	B6_30	South Africa	River	Draft	4.72	61.4	34.6	239	-	99.9	0.58	[33]
DRR199536	BEC1-S17-ESBL-03	Japan	Sea water	Draft	4.36	61.8	166.3	64	-	99.9	0.58	
DRR199537	BEC1-S17-ESBL-04	Japan	Sea water	Draft	4.53	61.4	85.1	133	-	99.9	0.00	
SRR7796540	OLC2686	Canada	Wastewater	Draft	4.88	61.1	30.1	353	-	100.0	0.58	[34]
DRR416204	10BC1	Japan	Wastewater	Draft	4.75	61.3	77.2	193	-	99.9	0.58	
DRR416206	10BC3	Japan	Wastewater	Draft	4.62	61.6	47.4	234	-	99.9	0.00	
DRR416209	10BC6	Japan	Wastewater	Draft	5.15	61.0	54.9	246	-	99.9	0.58	
DRR416212	10BC9	Japan	Wastewater	Draft	4.67	61.5	43.7	251	-	99.9	0.00	
DRR416262	10CC10	Japan	Wastewater	Draft	4.78	60.8	22.0	471	-	100.0	0.00	
DRR416260	10CC8	Japan	Wastewater	Draft	4.62	61.5	55.2	227	-	99.9	0.00	
DRR416184	12BC1	Japan	Wastewater	Draft	4.74	61.3	72.5	200	-	99.9	0.58	
DRR416188	2BC3	Japan	Wastewater	Draft	4.67	61.5	64.1	202	-	99.9	0.00	
DRR416222	2CC7	Japan	Wastewater	Draft	4.76	61.0	27.0	366	-	100.0	0.00	
DRR416223	2CC8	Japan	Wastewater	Draft	4.62	61.6	60.2	212	-	99.9	0.00	
DRR416191	4BC2	Japan	Wastewater	Draft	4.44	61.8	63.5	159	-	99.9	0.00	
DRR416235	4CC12	Japan	Wastewater	Draft	4.79	61.0	27.0	402	-	100.0	0.00	
DRR416228	4CC5	Japan	Wastewater	Draft	4.61	61.6	56.0	210	-	99.9	0.00	
DRR416229	4CC6	Japan	Wastewater	Draft	4.66	61.5	56.0	212	-	99.9	0.00	
DRR416231	4CC8	Japan	Wastewater	Draft	4.68	61.5	71.2	195	-	99.9	0.00	
DRR416232	4CC9	Japan	Wastewater	Draft	4.97	60.6	28.6	402	-	100.0	0.58	
DRR416196	6BC4	Japan	Wastewater	Draft	4.66	61.5	44.1	258	-	99.9	0.00	
DRR416198	6BC6	Japan	Wastewater	Draft	4.63	61.1	47.4	216	-	99.9	0.88	
DRR416199	6BC8	Japan	Wastewater	Draft	4.55	61.6	57.3	238	-	99.9	0.00	
DRR416246	6CC12	Japan	Wastewater	Draft	4.61	61.5	54.8	223	-	99.9	0.00	
DRR416240	6CC6	Japan	Wastewater	Draft	4.67	61.4	64.7	214	-	99.9	0.00	
DRR416242	6CC8	Japan	Wastewater	Draft	4.96	60.9	58.4	225	-	100.0	0.00	
DRR416202	8BC3	Japan	Wastewater	Draft	4.62	61.6	42.8	239	-	99.9	0.00	

DRR416203	8BC4	Japan	Wastewater	Draft	5.00	60.9	68.9	204	-	100.0	0.58
DRR416251	8CC4	Japan	Wastewater	Draft	5.01	60.8	58.4	233	-	100.0	0.00
DRR199174	WP2-S17-ESBL-01	Japan	Wastewater	Draft	4.41	61.9	178.7	58	-	99.9	0.00
DRR199202	WP2-S18-ESBL-02	Japan	Wastewater	Draft	4.76	61.2	27.4	406	-	99.9	0.58
DRR199204	WP2-S18-ESBL-04	Japan	Wastewater	Draft	4.43	61.4	46.6	196	-	99.3	0.00
DRR199210	WP2-S18-ESBL-10	Japan	Wastewater	Draft	4.67	60.9	25.0	390	-	100.0	0.00
DRR199187	WP2-W18-ESBL-02	Japan	Wastewater	Draft	4.53	61.4	30.3	374	-	99.9	0.00
DRR199188	WP2-W18-ESBL-03	Japan	Wastewater	Draft	4.70	61.3	58.3	194	-	99.9	0.58
DRR199221	WP2-W19-ESBL-07	Japan	Wastewater	Draft	4.80	60.7	26.6	379	-	100.0	0.58
DRR199245	WP3-W18-ESBL-03	Japan	Wastewater	Draft	4.73	61.0	23.8	408	-	100.0	0.58
DRR199311	WP4-S18-ESBL-01	Japan	Wastewater	Draft	4.49	61.4	35.8	277	-	99.9	1.17
DRR199315	WP4-S18-ESBL-05	Japan	Wastewater	Draft	4.46	61.5	28.8	310	-	99.9	1.17
DRR199320	WP4-S18-ESBL-10	Japan	Wastewater	Draft	4.34	61.8	53.3	158	-	99.9	0.00
DRR199298	WP4-W18-ESBL-02	Japan	Wastewater	Draft	4.73	61.3	30.2	360	-	99.9	0.88
DRR199329	WP4-W19-ESBL-06	Japan	Wastewater	Draft	4.46	61.8	38.8	251	-	99.9	0.00
DRR199338	WP5-S17-ESBL-06	Japan	Wastewater	Draft	4.86	60.7	28.1	376	-	100.0	0.58
DRR199339	WP5-S17-ESBL-07	Japan	Wastewater	Draft	4.96	60.6	26.1	457	-	100.0	0.00
DRR199366	WP5-S18-ESBL-03	Japan	Wastewater	Draft	4.46	61.5	42.4	248	-	99.9	0.00
DRR199351	WP5-W18-ESBL-03	Japan	Wastewater	Draft	4.31	61.8	107.4	90	-	99.9	0.00
DRR199352	WP5-W18-ESBL-04	Japan	Wastewater	Draft	4.67	61.1	31.0	331	-	100.0	0.00
DRR199393	WP7-S17-ESBL-07	Japan	Wastewater	Draft	4.63	61.7	165.4	62	-	99.9	0.00
DRR199407	WP7-S18-ESBL-01	Japan	Wastewater	Draft	4.46	61.6	55.3	178	-	99.9	0.58
DRR199408	WP7-S18-ESBL-02	Japan	Wastewater	Draft	4.57	61.6	78.6	141	-	99.9	0.00
DRR199409	WP7-S18-ESBL-03	Japan	Wastewater	Draft	4.38	61.6	34.2	286	-	99.9	0.00
DRR199398	WP7-W18-ESBL-01	Japan	Wastewater	Draft	4.68	61.2	32.4	293	-	99.9	0.58
DRR199429	WP8-S17-ESBL-01	Japan	Wastewater	Draft	4.29	61.8	29.5	294	-	99.9	0.00
DRR199430	WP8-S17-ESBL-02	Japan	Wastewater	Draft	4.29	61.8	32.3	289	-	99.9	0.00
DRR199437	WP8-S17-ESBL-09	Japan	Wastewater	Draft	4.56	61.6	39.1	241	-	99.9	0.00

DRR199461	WP8-S18-ESBL-03	Japan	Wastewater	Draft	4.34	61.6	29.2	306	-	99.9	0.00	
DRR199463	WP8-S18-ESBL-05	Japan	Wastewater	Draft	4.80	60.9	21.2	493	-	99.9	0.58	
DRR199467	WP8-S18-ESBL-09	Japan	Wastewater	Draft	4.80	61.1	47.4	241	-	99.9	0.00	
DRR199447	WP8-W18-ESBL-02	Japan	Wastewater	Draft	4.50	61.6	62.3	178	-	99.9	0.00	
DRR199497	WP9-S17-ESBL-02	Japan	Wastewater	Draft	4.36	61.8	133.3	76	-	99.9	0.58	
DRR199499	WP9-S17-ESBL-04	Japan	Wastewater	Draft	4.36	61.8	116.6	83	-	99.9	0.58	
DRR199517	WP9-S18-ESBL-02	Japan	Wastewater	Draft	4.33	61.6	38.4	237	-	99.3	0.00	
SRR22721361	ESBL07	New Zealand	Wastewater	Draft	4.68	60.9	25.8	379	-	100.0	0.58	
SRR18370014	FF6211022	USA	Wastewater	Draft	4.73	61.3	49.1	240	-	99.9	0.00	
SRR18369991	TA4211022	USA	Wastewater	Draft	4.51	61.7	333.3	54	-	99.9	0.00	
SRR12761640	tcs_102717_10	USA	Wastewater	Draft	4.65	61.4	32.3	273	-	99.9	0.00	[35]

A table of all *A. caviae* strains retrieved from the NCBI Assembly and SRA database analysed in this study. Completeness and contamination percentages were produced by the programme CheckM to assess genome quality.

References

1. **Giannattasio-Ferraz S, Maskeri L, Oliveira AP, Barbosa-Stancioli EF, Putonti C**. Draft Genome Sequence of *Aeromonas caviae* UFMG-H8, Isolated from Urine from a Healthy Bovine Heifer (Gyr Breed). *Microbiology Resource Announcements* 2020;9(19):10.1128/mra.00388-00320.

2. **Boehmer T, Vogler AJ, Thomas A, Sauer S, Hergenroether M et al.** Phenotypic characterization and whole genome analysis of extended-spectrum beta-lactamase-producing bacteria isolated from dogs in Germany. *PLOS ONE* 2018;13(10):e0206252.

3. **Li R, Chan EW-c, Chen S**. Characterisation of a chromosomally-encoded extended-spectrum β-lactamase gene $bla_{PER\text{-}3}$ in *Aeromonas caviae* of chicken origin. *International Journal of Antimicrobial Agents* 2016;47(1):103-105.

4. **Chan K-G, Chin P-S, Tee KK, Chang C-Y, Yin W-F et al.** Draft Genome Sequence of *Aeromonas caviae* Strain L12, a Quorum-Sensing Strain Isolated from a Freshwater Lake in Malaysia. *Genome Announcements* 2015;3(2):10.1128/genomea.00079-00015.

5. **Cairns J, Jokela R, Hultman J, Tamminen M, Virta M et al.** Construction and Characterization of Synthetic Bacterial Community for Experimental Ecology and Evolution. *Frontiers in Genetics*, Methods 2018;9.

6. **Hu X, Zhang H, Liu Y, Liu X, Qiao J et al.** Genetic characterization and virulence determinants of multidrug-resistant NDM-1-producing *Aeromonas caviae*. *Frontiers in Microbiology*, Original Research 2023;13.

7. **Luo X, Yin Z, Yu L, Zhang J, Hu D et al.** Genomic analysis of chromosomal cointegrated $bla_{NDM\text{-}1}$-carrying ICE and $bla_{RSA\text{-}1}$-carrying IME from clinical multidrug resistant *Aeromonas caviae*. *Frontiers in Cellular and Infection Microbiology*, Original Research 2023;13.

8. **Pérez-Valdespino A, Fernández-Rendón E, Curiel-Quesada E**. Detection and characterization of class 1 integrons in *Aeromonas* spp. isolated from human diarrheic stool in Mexico. *Journal of Basic Microbiology* 2009;49(6):572-578.

9. **Moriel B, Cruz LM, Dallagassa CB, Faoro H, Souza EMd et al.** Draft Genome Sequence of *Aeromonas caviae* 8LM, Isolated from Stool Culture of a Child with Diarrhea. *Genome Announcements* 2015;3(3):e00524-00515.

10. **Sichtig H, Minogue T, Yan Y, Stefan C, Hall A et al.** FDA-ARGOS is a database with public quality-controlled reference genomes for diagnostic use and regulatory science. *Nature Communications* 2019;10(1):3313.

11. **Tang L, Huang J, She J, Zhao K, Zhou Y**. Co-Occurrence of the bla (KPC-2) and Mcr-3.3 Gene in *Aeromonas caviae* SCAc2001 Isolated from Patients with Diarrheal Disease. *Infection and Drug Resistance* 2020;13:1527-1536.

12. **Gray K, Green LR, Chaudhuri RR, Shaw JG**. Draft Whole-Genome Sequences of 10 *Aeromonas* Strains from Clinical and Environmental Sources. *Microbiology Resource Announcements* 2019;8(30):10.1128/mra.00170-00119.

13. **Anandan S, Gopi R, Devanga Ragupathi NK, Muthuirulandi Sethuvel DP, Gunasekaran P et al.** First report of *bla*OXA-181-mediated carbapenem resistance in *Aeromonas caviae* in association with pKP3-A: Threat for rapid dissemination. *Journal of Global Antimicrobial Resistance* 2017;10:310-314.

14. **Talagrand-Reboul E, Colston SM, Graf J, Lamy B, Jumas-Bilak E**. Comparative and Evolutionary Genomics of Isolates Provide Insight into the Pathoadaptation of *Aeromonas*. *Genome Biology and Evolution* 2020;12(5):535-552.

15. **Bertran X, Rubio M, Gómez L, Llovet T, Muñoz C et al.** Taxonomic Identification of Different Species of the Genus *Aeromonas* by Whole-Genome Sequencing and Use of Their Species-Specific β-Lactamases as Phylogenetic Markers. *Antibiotics* 2021;10(4):354.

16. **Shen Y, Xu C, Sun Q, Schwarz S, Ou Y et al.** Prevalence and Genetic Analysis of *mcr*-3-Positive *Aeromonas* Species from Humans, Retail Meat, and Environmental Water Samples. *Antimicrobial Agents and Chemotherapy* 2018;62(9):10.1128/aac.00404-00418.

17. **Luo X, Mu K, Zhao Y, Zhang J, Qu Y et al.** Emergence of *bla*NDM– 1-Carrying *Aeromonas caviae* K433 Isolated From Patient With Community-Acquired Pneumonia. *Frontiers in Microbiology*, Original Research 2022;13.

18. **Padilla JCA, Bustos P, Castro-Escarpulli G, Sánchez-Varela A, Palma-Martinez I et al.** Draft Genome Sequence of *Aeromonas caviae* Strain 429865 INP, Isolated from a Mexican Patient. *Genome Announcements* 2015;3(5):10.1128/genomea.01240-01215.

19. **Dubey S, Ager-Wick E, Kumar J, Karunasagar I, Karunasagar I et al.** *Aeromonas* species isolated from aquatic organisms, insects, chicken, and humans in India show similar antimicrobial resistance profiles. *Frontiers in Microbiology*, Original Research 2022;13.

20. **Coleman-Derr D**. *Exploring the role of drought-induced plant associated microbes in promoting plant fitness in Sorghum bicolor and Oryza sativa*. United States2016.

21. **Silva LCAd, Leal-Balbino TC, Melo BSTd, Mendes-Marques CL, Rezende AM et al.** Genetic diversity and virulence potential of clinical and environmental *Aeromonas* spp. isolates from a diarrhea outbreak. *BMC Microbiology* 2017;17(1):179.

22. **Cardozo FA, Vargas NCA, Zimpel CK, Pessoa A, Rivera ING**. Draft Genome Sequence of *Aeromonas caviae* CH129, a Marine-Derived Bacterium Isolated from the Coast of São Paulo State, Brazil. *Genome Announcements* 2016;4(6):10.1128/genomea.01336-01316.

23. **Chen C, Chen L, Zhang Y, Cui C-Y, Wu X-T et al.** Detection of chromosome-mediated *tet*(X4)-carrying *Aeromonas caviae* in a sewage sample from a chicken farm. *Journal of Antimicrobial Chemotherapy* 2019;74(12):3628-3630.

24. **Mathys DA, Mollenkopf DF, Feicht SM, Adams RJ, Albers AL et al.** Carbapenemase-producing *Enterobacteriaceae* and *Aeromonas* spp. present in wastewater treatment plant effluent and nearby surface waters in the US. *PLOS ONE* 2019;14(6):e0218650.

25. **Sekizuka T, Inamine Y, Segawa T, Hashino M, Yatsu K et al.** Potential KPC-2 carbapenemase reservoir of environmental *Aeromonas hydrophila* and *Aeromonas caviae* isolates from the effluent of an urban wastewater treatment plant in Japan. *Environmental Microbiology Reports* 2019;11(4):589-597.

26. **Li Y, Qiu Y, Fang C, Dai X, Zhang L**. Genomic Characterization of a Multidrug-Resistant *Aeromonas caviae* Isolate Carrying a Novel $bla_{\mathrm{KPC-2}}$-Harbouring Plasmid and an IMP-4-Encoding Phage-like Plasmid. *Microbiology Spectrum* 2022;10(4):e00840-00822.

27. **Jałowiecki Ł, Płaza G, Nowrotek M**. Whole-Genome Sequences of Antibiotic-Resistant *Aeromonas caviae* Strains Isolated from Treated Wastewater. *Microbiology Resource Announcements* 2020;9(40).

28. **Shi Y, Tian Z, Leclercq SO, Zhang H, Yang M et al.** Genetic characterization and potential molecular dissemination mechanism of *tet*(31) gene in *Aeromonas caviae* from an oxytetracycline wastewater treatment system. *Journal of Environmental Sciences* 2019;76:259-266.

29. **Rangel LT, Marden J, Colston S, Setubal JC, Graf J et al.** Identification and characterization of putative *Aeromonas* spp. T3SS effectors. *PLOS ONE* 2019;14(6):e0214035.

30. **Cardozo FA, Zimpel CK, Guimaraes AMS, Pessoa A, Rivera ING**. Draft Genome Sequence of Marine-Derived *Aeromonas caviae* CHZ306, a Potential Chitinase Producer Strain. *Genome Announcements* 2016;4(6):10.1128/genomea.01293-01216.

31. **Poole TL, Schlosser WD, Crippen TL, Swiger SL, Norman KN et al.** Whole-Genome Sequence of *Aeromonas* spp. Isolated from a Dairy Farm in Central Texas. *Microbiology Research* 2023;14(1):161-176.

32. **Song Y, Wang LF, Zhou K, Liu S, Guo L et al.** Epidemiological characteristics, virulence potential, antimicrobial resistance profiles, and phylogenetic analysis of *Aeromonas caviae* isolated from extra-intestinal infections. *Frontiers in Cellular and Infection Microbiology* 2023;13:1084352.

33. **Tucker K, Mageiros L, Carstens A, Bröcker L, Archer E et al.** Spatiotemporal Investigation of Antibiotic Resistance in the Urban Water Cycle Influenced by Environmental and Anthropogenic Activity. *Microbiology Spectrum* 2022;10(5):e02473-02422.

34. **Cooper AL, Carter C, McLeod H, Wright M, Sritharan P et al.** Detection of carbapenem-resistance genes in bacteria isolated from wastewater in Ontario. *FACETS* 2021;6:569-591.

35. **Gray HK, Arora-Williams KK, Young C, Bouwer E, Davis MF et al.** Contribution of Time, Taxonomy, and Selective Antimicrobials to Antibiotic and Multidrug Resistance in Wastewater Bacteria. *Environmental Science & Technology* 2020;54(24):15946-15957.
